# One-Pot Three-Component Synthesis of Novel Diethyl((2-oxo-1,2-dihydroquinolin-3-yl)(arylamino)methyl)phosphonate as Potential Anticancer Agents

**DOI:** 10.3390/ijms17050653

**Published:** 2016-04-29

**Authors:** Yi-Lin Fang, Zhi-Lin Wu, Meng-Wu Xiao, Yu-Ting Tang, Kang-Ming Li, Jiao Ye, Jian-Nan Xiang, Ai-Xi Hu

**Affiliations:** 1 College of Chemistry and Chemical Engineering, Hunan University, Changsha 410082, China; b12110055@hnu.edu.cn (Y.-L.F.); wuzhilin@hnu.edu.cn (Z.-L.W.); mwxiao@hnu.edu.cn (M.-W.X.); tangyuting14@hnu.edu.cn (Y.-T.T.); ming11325@hnu.edu.cn (K.-M.L.); yejiao@hnu.edu.cn (J.Y.); jnxiang@hnu.edu.cn (J.-N.X.); 2 College of Food and Biochemical Engineering, Gaungxi Science & Technology Normal University, Laibin 546199, China

**Keywords:** 2-oxoquinoline, α-aminophosphonate, one-pot method, anticancer activity, apoptosis

## Abstract

With the aim of discovering new anticancer agents, we have designed and synthesized novel α-aminophosphonate derivatives containing a 2-oxoquinoline structure using a convenient one-pot three-component method. The newly synthesized compounds were evaluated for antitumor activities against the A549 (human lung adenocarcinoma cell), HeLa (human cervical carcinoma cell), MCF-7 (human breast cancer cell), and U2OS (human osteosarcoma cell) cancer cell lines *in vitro*, employing a standard 3-(4,5-dimethyl-2-thiazolyl)-2,5-diphenyl-2-*H*-tetrazolium bromide (MTT) assay. The results of pharmacological screening indicated that many compounds exhibited moderate to high levels of antitumor activities against the tested cancer cell lines and that most compounds showed more potent inhibitory activities comparable to 5-fluorouracil (5-FU) which was used as a positive control. The mechanism of representative compound **4u** (diethyl((2-oxo-1,2-dihydroquinolin-3-yl)(phenyl-amino)methyl)phosphonate) indicated that the compound mainly arrested HeLa cells in S and G2 stages and was accompanied by apoptosis in HeLa cells. This action was confirmed by acridine orange/ethidium bromide staining, Hoechst 33342 staining, and flow cytometry.

## 1. Introduction

Malignant cancer is the primary cause of human death worldwide [1], accounting for 8.2 million deaths in 2012, and it is expected that annual cases will rise from 14 million in 2012 to 22 within the next two decades [2,3]. During the last few decades, researchers have struggled to find effective clinical approaches for the treatment of cancer and have searched for novel anticancer agents, but the management of malignancies in humans still constitutes a major concern for contemporary medicine [4,5,6,7]. Therefore, it is urgent to discover new anticancer agents of novel chemical entity.

**Scheme 1:**
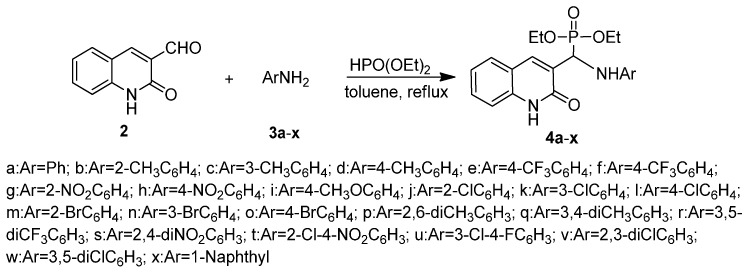
Synthesis of **4a**–**x** compounds.

2-Oxoquinolines (**1**, Scheme 1), a class of heterocyclic molecular scaffold, received much interest and attention due to their diverse pharmacological activities, which include antimicrobial [8], anti-angiogenic [9], anticancer [10], antioxidant [11], antimalarial [12], and anti-inflammatory [13,14] properties. The 2-oxoquinoline-3-carbaldehyde (**2**, Scheme 1), which is an interesting building block, occupies a prominent position as a key intermediate for further annelation and for various functional group interconversions [15,16]. A recent investigation indicated that 2-oxoquinoline displays enhanced activity towards cancer cell lines [17,18]. Therefore, the active pharmacal core of 2-oxoquinoline was chosen to screen for new potential antitumor compounds by the introduction of different functional groups.

The literature search that was carried out identified that the aminophosphonate group is considered a potent antitumor agent and is able to effectively enhance antitumor activity by introducing a pharmacy core [19]. α-Aminophosphonate derivatives have received increasing attention in recent years due to their wide range of applications in organic and medicinal chemistry. He *et al.* [20] found that artesunate α-aminophosphonate derivatives have promising antimicrobial activities. Devarayan *et al.* [21] reported α-aminophosphonate chitosan derivatives as antifungal agents against *Aspergillus niger*. Abdou *et al.* [22] studied the synthesis of enamino and α-aminophosphonates as peptidomimetics of analgesic/anti-inflammatory. Moreover, α-aminophosphonates have broad biological activities and many aminophosphonates derivatives displayed good anticancer activities in recent research [23,24,25,26]. Huang *et al.* [23] reported a series of α-aminophosphonates dehydroabietic acid derivatives and their antitumor activities. Ye *et al.* [24] synthesized novel alizarin α-aminophosphonate derivatives and evaluated their biological activities as anticancer agents. Li *et al.* [25] investigated that the introduction of aminophosphonates groups into coumarin can enhance bioactivity as antitumor agents. Foroogh *et al.* [26] reported the synthesis of new α-aminophosphonate derivatives incorporating benzimidazole, theophylline, and adenine nucleobases and evaluated their anticancer properties.

**Figure 1:**
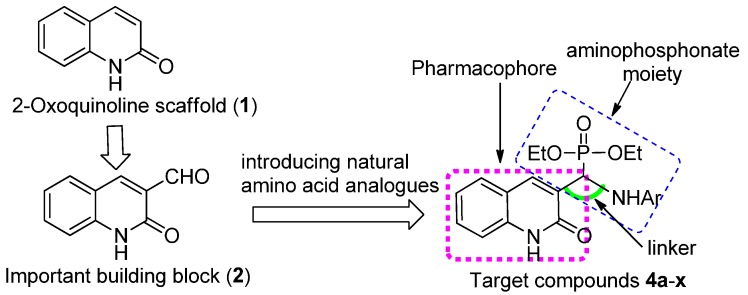
Design of novel α-aminophosphonates derivatives as anticancer agents.

Due to the synthetic and biological values of 2-oxoquinoline and aminophosphonate groups, we expect that the incorporation of aminophosphonate and 2-oxoquinoline groups may lead to good antitumor activity. However, to the best of our knowledge, the studies on the synthesis, antitumor activities, and apoptosis-inducing effects of α-aminophosphonate derivatives derivated from 2-oxoquinoline have been little explored to date. Accordingly, the aminophosphonate group was rationally designed and introduced to 2-oxoquinoline as anticancer agents (Figure 1).

In this paper, our present work is to design and synthesize α-aminophosphonates derivatives containing a 2-oxoquinoline skeleton, and investigate their potential anticancer activity against four human cancer cell lines. The overall strategies for the synthesis of **4a**–**x** are outlined in Scheme 1. Preliminary research on the mode of action of representative compound **4u** is also investigated.

## 2. Results and Discussion

### 2.1. Biological Activity

#### 2.1.1. *In Vitro* Cytotoxic Activity

The *in vitro* cytotoxic potency of 2-oxo-quinoline aminophosphonate derivatives **4a**–**x** were evaluated by MTT assay against A549, HeLa, MCF-7 and U2OS cancer cell lines with 5-fluorouracil (5-FU) as the positive control. The IC_50_ values (µM) (concentration required to achieve 50% inhibition of the tumor cell proliferation) of the tested compounds for each cell line are presented in Table 1.

As shown in Table 1, all the **4** compounds except **4o** showed anticancer activities in the A549 assay. The results showed that all compounds exhibited better inhibition than 2-oxo-quinoline (IC_50_ > 200 µM) except the compound **4o** in the A549 assay, and compounds **4j**, **4q**, **4r**, **4s**, **4u**, and **4x** exhibited better inhibition than the positive control (5-FU, IC_50_ = 34.32 ± 3.4 µM), with IC_50_ in the range of 16.6 ± 0.9–31.1 ± 0.1 µM. Additionally, the screening results indicated that the introduction of an aminophosphonate group to 2-oxo-quinoline should obviously improve the anticancer activity against the A549 cell line. Compound **4u** exhibited the best anticancer activity out of all the compounds, with IC_50_ of 16.6 ± 0.9 µM, while compound **4o** showed the lowest, with IC_50_ > 200 µM. On the basis of the above observation, the substituents in benzene group of aminophosphonate moiety possess significant effects on the cytotoxic inhibition in the A549 assay, and the introduction of electron donor substituents may result in lower cytotoxic inhibition compared with withdrawing electron substituents, except for compounds **4h**, **4l**, and **4o**. In addition, the chloro group at the *ortho* and *meta* positions of the benzene group may possess important effects on the anticancer activity, while the chloro group at the *para* position exhibited a negative effect. Additionally, disubstituted groups of benzene also have important effects for the anticancer activities in the A549 assay, and the introduction of fluorine and halogen groups may lead to the enhancement of cytotoxic inhibition. Likewise, the compound **4x** (with IC_50_ of 29.4 ± 0.5 µM), in which the naphthalene replaced benzene, also showed important influences on the anticancer activity in this assay.

In the HeLa assay, with the exception of the compound **4o**, all the **4** compounds exhibited much better inhibition than 2-oxo-quinoline (IC_50_ > 200 µM), with IC_50_ in the range of 2.5 ± 0.6–137.0 ± 8.9 µM. The screening results indicated that the introduction of α-aminophosphonate to 2-oxo-quinoline markedly increased the anticancer activity against the HeLa cell line. Among all the compounds, compound **4u** exhibited the best inhibitory activity, with IC_50_ of 2.5 ± 0.6 µM, while compound **4o** showed the lowest, with IC_50_ > 200 µM. Through the above observations, it could be summarized that monosubstituted groups of methyl group at the *ortho* and *meta* positions, trifluoromethyl at the *meta* and *para* positions, and bromine at the *meta* position in benzene group may have important influences on the cytotoxicity. Additionally, disubstituted groups in benzene also have crucial effects on the anticancer activities in the HeLa assay, except for **4w** the introduction of withdrawing electron groups may lead to the enhancement of cytotoxic inhibition, and the introduction of electron donor groups may lead to the exhibited negative effect. Also, the naphthalene replaced benzene, which proved to have important influences on the anticancer activity in this assay. The compound **4x** has better cytotoxicity with IC_50_ of 27.4 ± 6.0 µM.

In the MCF-7 assay, with the exception of compound **4o**, all compounds showed better antitumor activities than 2-oxo-quinoline (IC_50_ > 200 µM), with IC_50_ in the range of 0.3 ± 0.1–174.3 ± 8.6 µM, thereby showing that the combination of α-aminophosphonate and 2-oxo-quinoline would enhance the cytotoxicities against the MCF-7 cell line. Moreover, with the exception of compounds **4a**, **4c**, **4g**, **4o**, and **4p**, other compounds even demonstrated better cytotoxicities than 5-FU, indicating favorable antitumor activities of these compounds toward the MCF-7 cell line. When compared with other compounds, compounds **4u** and **4x** showed better inhibition on the MCF-7 cell line, with IC_50_ of 1.0 ± 0.4 and 0.3 ± 0.1 µM, respectively. Evidently, methyl and methoxy at the *para* position and trifluoromethyl at the *meta position*, as well as chlorine and bromine at the *ortho* position in the benzene group may result in the improvement of antitumor activity, while the presence of methyl at the *meta* position and nitro at the *ortho* position in benzene cause the decrease of cytotoxicity. In addition, disubstituted groups of 3-Cl-4-F in benzene and replacement of benzene with naphthalene also showed important influences on the cytotoxicity in this assay.

In the U2OS assay, all the compounds except **4a** and **4d** displayed better cytotoxicity than 2-oxo-quinoline (IC_50_ > 200 µM), with IC_50_ in the range of 2.5 ± 0.2–157.6 ± 4.1 µM, showing that the combination of α-aminophosphonate and 2-oxo-quinoline would enhance the cytotoxicity against the U2OS cell line. It should be noted that seven compounds (**4n**, **4p**, **4t**, **4u**, **4v**, **4w**, and **4x**) exhibited better cytotoxic inhibition, with IC_50_ values lower than 50 µM, thereby showing good inhibitory activities on the U2OS cell line. Among all the compounds, compound **4x** exhibited the best inhibitory activity against the U2OS cells, with IC_50_ of 2.5 ± 0.2 µM, while compounds **4a** and **4d** displayed the lowest, with IC_50_ > 200 µM. Obviously, the results indicated that the bromine at *meta* position and disubstituted groups of 2,6-diCH_3_, 2-Cl-4-NO_2_, 3-Cl-4-F, 2,3-diCl, and 3,5-diCl in the benzene group were important contributors to the antitumor activity. Likewise, the compound **4x**, in which the naphthalene replaced benzene, showed good inhibitory activities against the U2OS cell line and also displayed important influences on the cytotoxicity in this assay.

In our present study, compound **4u**, which displayed good cytotoxic inhibition in four cell lines and could be treated as a favorable representative of compounds **4**, was selected to evaluate its mechanism of growth inhibition on HeLa cells by acridine orange(AO)/ethidium bromide(EB) staining, Hoechst 33324 staining, and flow cytometry.

#### 2.1.2. Apoptosis Assessment by Acridine Orange(AO)/Ethidium Bromide(EB) Staining

**Figure 2:**
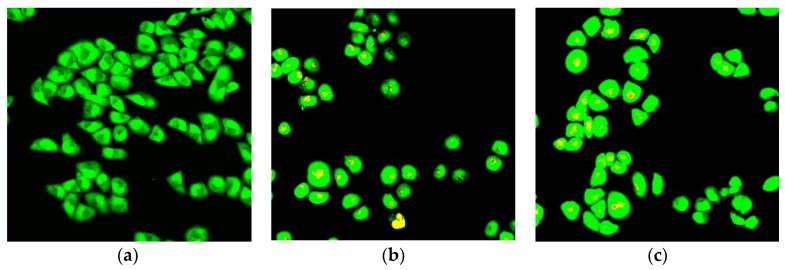
Acridine orange(AO)/ethidium bromide(EB) staining of compound **4u** in HeLa cells. (**a**) Cells that were not treated with compound **4u** were used as a control for 24 h; (**b**,**c**) cells that were treated with compound **4u** (10 µM) for 12 and 24 h, respectively. The eyepiece was working at 20×.

AO and EB are important dyes. AO can pass through an intact cell membrane, then stain nuclear DNA, while EB only stains cells with damaged cell membranes. Therefore, after being simultaneously treated with AO and EB, normal cells will be stained as green, early apoptotic cells will be stained as green-yellow or display green-yellow fragments, Ad late apoptotic cells will be thickly stained as orange or show orange fragments, and non-apoptotic cells will be stained as orange-yellow. The cytotoxicity of compound **4u** at the concentration of 10 µM against HeLa cells from 12 to 24 h was tested by AO/EB staining, and HeLa cells not treated with the **4u** were used as a control for 24 h. The results are displayed in Figure 2. As shown in Figure 2, the HeLa cells treated with **4u** from 12 to 24 h, at the concentration of 10 µM, had evidently changed. The nuclei obviously stained evenly as yellow-green or light orange, and the cell morphology exhibited pycnosis, membrane blebbing, and cell budding. These phenomena were related to cell apoptosis. On the basis of the above observation, the cells displayed an apoptotic morphology. These results demonstrated that compound **4u** could cause the decrease of apoptosis with low cytotoxicity.

#### 2.1.3. Apoptosis Assessment by Hoechst 33342 Staining

**Figure 3:**
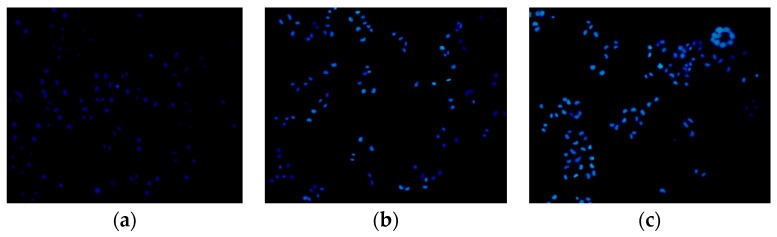
Hoechst 33342 staining of compound **4u** in Hela cells. (**a**) Cells that were not treated with compound **4u** were used as a control for 12 h; (**b**,**c**) cells that were treated with compound **4u** (5, and 10 µM) for 12 h, respectively. The eyepiece was working at 10×.

Hoechst 33342, which is a membrane permeable dye, can stain the cell nucleus with blue fluorescence. Normal cells with equably light blue nuclei were evidently detected by a fluorescence microscope after dyeing with Hoechst 33342, while apoptotic cells nuclei were stained bright blue because of karyopyknosis and chromatin condensation. However, the nuclei of the dead cells could not be stained. HeLa cells treated with compound **4u** at 5 and 10 µM for 12 h were stained with Hoechst 33342. HeLa cells not treated with the **4u** were used as a control for 12 h. The results were given in Figure 3. As shown in Figure 3, cells not treated with compound **4u** were normally blue (in the web version). It should be noted that for the **4u** treatment the cells exhibited strong blue fluorescence and demonstrated typical apoptotic morphology after 12 h and simultaneous changes in fluorescence due to the change of concentration. The observation indicated that compound **4u** induced apoptosis against Hela cell lines, consistent with the results of AO/EB double staining.

#### 2.1.4. Apoptosis Study by Flow Cytometry Assay

**Figure 4:**
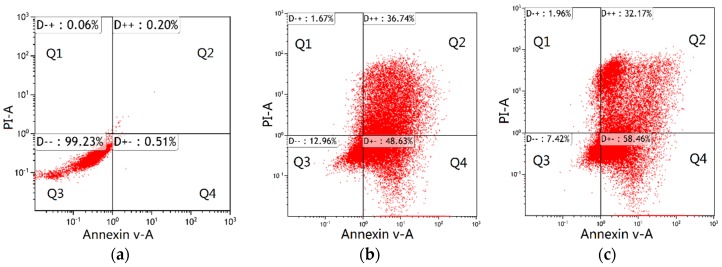
Apoptosis ratio detection of compound **4u** by Annexin V-FITC (Fluorescein Isothiocyanate)/PI (Propidium Iodide) assay. (**a**) HeLa cells were not treated with **4u** for 12 h; (**b**,**c**) HeLa cells were treated with compound **4u** at 10 and 15 µM for 12 h, respectively. D-+: FITC-/PI+; D++: FITC+/PI+; D--: FITC-/PI-; D+-: FITC+/PI-.

The apoptosis ratios induced by compound **4u** in HeLa tumor cells were quantitatively evaluated by flow cytometry. Four quadrant images were obtained by flow cytometric analysis: the damaged cells and the necrotic cells were showed in the Q1 area, the later period apoptotic cells were located in the Q2 region, the normal cells were represented in the Q3 area, and the early apoptotic cells were displayed in the Q4 region. The results were given in Figure 4.

As shown in Figure 4 and Table 2, the apoptosis ratios (late plus early apoptotic cells, Q2 + Q4 quadrants) for compound **4u** were significantly increased within the initial 12 h of treatment at the concentration of 10 and 15 µM. The apoptosis of HeLa cells treated with compound **4u** increased gradually in a concentration manner. The apoptosis ratios of compound **4u** were measured at different concentration points and were found to be 85.37% (10 µM) and 90.63% (15 µM), while the apoptosis ratio for the control was 0.71%. These results suggested that compound **4u** can suppress cell proliferation by inducing apoptosis.

#### 2.1.5. Investigation of Cell Cycle Distribution

**Figure 5:**
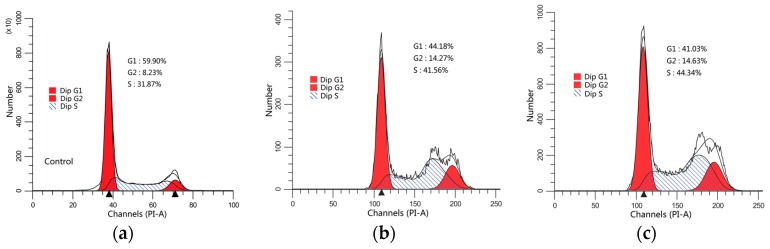
Cell cycle analysis of compound **4u** by flow cytometry. (**a**) HeLa cells were not treated with **4u** for 12 h; (**b**,**c**) HeLa cells were treated with compound **4u** at 5 and 10 µM for 12 h, respectively.

Cell cycle distribution in HeLa cells was examined to determine whether or not compound **4u** inhibits the proliferation of these cells through cell cycle arrest. The effect of compounds **4u** on the cell cycle of HeLa cell lines was evaluated by flow cytometry at 5 and 10 µM for 12 h and shown in Figure 5 and Table 3. Cell cycle analysis demonstrated that compound **4u** treatment increased the population of cells in the S and G2 phases on a concentration dependent basis. This phenomenon was accompanied by a decrease in the population of cells in the G1 phase compared with the negative control. As shown in Table 3, the population of HeLa cells in the G0/G1 phase decreased from 59.90% (control) to 41.03%, and in the S phase and G2 phase increased from 31.87% and 8.23% (control) to 44.34% and 14.63%, respectively, after treatment with 10 µM of the **4u** compound for 12 h. These results indicated that cell cycle arrest in the both S and G2 stages contributed to the antiproliferative effects of compound **4u** on HeLa cells.

## 3. Experimental Section

### 3.1. Chemistry

All chemicals and solvents were analytical reagents and used directly without further depuration. Melting points were assessed by an X-4 electrothermal digital melting point (Beijing Taike Instruments Co., Ltd., Beijing, China) apparatus and were uncorrected. Nuclear magnetic resonance (NMR) was recorded on a Varian INOVA-400 spectrometer (Varian, Palo Alto, CA, USA) in dimethyl sulfoxide (DMSO-*d*_6_) or deuterated chloroform (CDCl_3_), whereas tetramethylsilane (TMS) (δ 0 ppm) was used as an internal standard for spectra recorded. Elemental analysis was carried out on an Elementar Vario EL CHNS Elemental Analyzer (Elementar, Hanau, Germany). Analyses for C, H, and N were within 0.4% of the theoretical values.

General procedure for the synthesis of **4a**–**x**. Compound **2** (2 mmol) and different aromatic amine (2 mmol) were added to toluene (15 mL). The mixture was stirred at room temperature for 15 min and diethyl phosphate was added to the mixture and then refluxed for 1–5 h. When completed, the solvent was evaporated in a vacuum, and the crude product was purified by silica gel chromatography using petroleum ether/ethyl acetate (*v:v* = 4:1) to afford compounds **4a**–**x**.

*Diethyl((2-oxo-1,2-dihydroquinolin-3-yl)(phenylamino)methyl)phosphonate* (**4a**)*.* Following the general procedure the compound **4a** was obtained as a white solid, yield 60.0%, m.p. 194–196 °C. ^1^H-NMR (400 MHz, CDCl_3_): δ 11.53 (s, 1H, NH), 8.06 (d, *J* = 3.7 Hz, 1H), 7.53 (t, *J* = 7.8 Hz, 1H), 7.49 (d, *J* = 7.7 Hz, 1H), 7.36 (d, *J* = 8.1 Hz, 1H), 7.19 (t, *J* = 7.9 Hz, 1H), 7.11 (t, *J* = 7.9 Hz, 2H), 6.74–6.68 (m, 3H), 5.58, 5.52 (s, 1H, N-CH-P), 4.30–4.25 (m, 2H, OCH_2_), 4.13–3.97 (m, 2H, OCH_2_), 1.34 (t, 3H, *J* = 7.1 Hz, CH_3_), 1.14 (t, 3H, *J* = 7.1 Hz, CH_3_). ^13^C-NMR (100 MHz, CDCl_3_): δ 163.43, 146.26, 138.66, 138.04, 130.53, 129.30, 128.66, 128.17, 122.69, 119.99, 118.47, 115.81, 113.74, 63.89, 63.52, 48.70 (47.16), 16.54, 16.30. Anal. calcd. for C_20_H_23_N_2_O_4_P: C, 62.17; H, 6.00; N, 7.25. Found C, 62.15; H, 5.98; N, 6.04.

*Diethyl((2-oxo-1,2-dihydroquinolin-3-yl)(o-tolylamino)methyl)phosphonate* (**4b**). Following the general procedure the compound **4b** was obtained as a light brown solid, yield 86.3%, m.p. 191–193 °C. ^1^H-NMR (400 MHz, CDCl_3_): δ 11.59 (s, 1H, NH), 8.01 (d, *J* = 3.6 Hz, 1H), 7.54 (d, *J* = 7.9 Hz, 1H), 7.49 (t, *J* = 7.6 Hz, 1H), 7.40 (d, J = 7.6 Hz, 1H), 7.20 (t, *J* = 7.4 Hz, 1H), 7.05 (d, *J* = 7.2 Hz, 1H), 6.97 (t, *J* = 7.7 Hz, 1H), 6.65 (t, *J* = 7.3 Hz, 1H), 6.58 (d, *J* = 8.0 Hz, 1H), 5.56, 5.50 (s, 1H, NCHP), 4.86 (s, 1H, NH), 4.32–4.23 (m, 2H, OCH_2_), 4.15–3.96 (m, 2H, OCH_2_), 2.30 (s, 3H, CH_3_), 1.34 (t, 3H, *J* = 7.0 Hz, CH_3_), 1.15 (t, 3H, *J* = 6.9 Hz, CH_3_). ^13^C-NMR (100 MHz, CDCl_3_): δ 163.37, 143.92, 138.35, 138.02, 130.58, 130.30, 128.49, 128.17, 127.23, 123.02, 122.78, 119.98, 118.31, 115.89, 111.00, 63.79, 63.53, 49.26 (47.71), 17.66, 16.54, 16.32. Anal. calcd. for C_21_H_25_N_2_O_4_P: C, 62.99; H, 6.29; N, 7.00. Found C, 62.96; H, 6.27; N, 7.04.

*Diethyl((2-oxo-1,2-dihydroquinolin-3-yl)(m-tolylamino)methyl)phosphonate* (**4c**). Following the general procedure the compound **4c** was obtained as a yellow solid, yield 77.5%, m.p. 174–176 °C. ^1^H-NMR (400 MHz, CDCl_3_): δ 11.37 (s, 1H, NH), 8.04 (d, *J* = 3.7 Hz, 1H), 7.53 (d, *J* = 7.8 Hz, 1H), 7.47 (t, *J* = 7.7 Hz, 1H), 7.34 (d, *J* = 8.2 Hz, 1H), 7.18 (t, *J* = 7.5 Hz, 1H), 6.99 (t, *J* = 7.7 Hz, 1H), 6.58–6.50 (m, 3H), 5.56–5.50 (m, 1H, NCHP), 4.29–4.25 (m, 2H, OCH_2_), 4.12–3.95 (m, 2H, OCH_2_), 2.20 (s, 3H, CH_3_), 1.34 (t, 3H, *J* = 7.1 Hz, CH_3_), 1.13 (t, 3H, *J* = 7.0 Hz, CH_3_). ^13^C-NMR (100 MHz, CDCl_3_): δ 163.40, 146.16, 139.08, 138.53, 137.97, 130.50, 129.19, 128.71, 128.16, 122.70, 119.97, 119.48, 115.82, 114.66, 110.59, 63.83, 63.47, 48.81 (47.28), 21.57, 16.53, 16.29. Anal. calcd. for C_21_H_25_N_2_O_4_P: C, 62.99; H, 6.29; N, 7.00. Found C, 63.01; H, 6.26; N, 6.99.

*Diethyl((2-oxo-1,2-dihydroquinolin-3-yl)(p-tolylamino)methyl)phosphonate* (**4d**). Following the general procedure the compound **4d** was obtained as a yellow solid, yield 72.5%, m.p. 181–184 °C. ^1^H-NMR (400 MHz, CDCl_3_): δ 11.47 (s, 1H, NH), 8.06 (d, *J* = 3.3 Hz, 1H), 7.52 (d, *J* = 7.9 Hz, 1H), 7.47 (t, *J* = 7.7 Hz, 1H), 7.34 (d, *J* = 8.2 Hz, 1H), 7.18 (t, *J* = 7.5 Hz, 1H), 6.93 (d, *J* = 8.2 Hz, 2H), 6.67 (d, *J* = 7.8 Hz, 2H), 5.55, 5.49 (s, 1H, NCHP), 4.32–4.24 (m, 2H, OCH_2_), 4.12–3.96 (m, 2H, OCH_2_), 2.17 (s, 3H, CH_3_), 1.34 (t, 3H, *J* = 7.1 Hz, CH_3_), 1.14 (t, 3H, *J* = 7.1 Hz, CH_3_). ^13^C-NMR (100 MHz, CDCl_3_): δ 163.37, 143.64, 138.67, 137.99, 130.51, 129.81, 128.60, 128.18, 127.90, 122.68, 119.98, 115.80, 114.02, 63.81, 63.45, 49.12 (47.58), 20.38, 16.48, 16.25. Anal. calcd. for C_21_H_25_N_2_O_4_P: C, 62.99; H, 6.29; N, 7.00. Found C, 62.98; H, 6.25; N, 7.02.

*Diethyl((2-oxo-1,2-dihydroquinolin-3-yl)((3-(trifluoromethyl)phenyl)amino)methyl)Phosphonate* (**4e**). Following the general procedure the compound **4e** was obtained as a yellow solid, yield 79.2%, m.p. 219–222 °C. ^1^H-NMR (400 MHz, CDCl_3_): δ 11.91 (s, 1H, NH), 8.10 (d, *J* = 3.6 Hz, 1H), 7.53 (d, *J* = 8.0 Hz, 1H), 7.45 (t, *J* = 8.0 Hz, 1H), 7.37 (d, *J* = 8.0 Hz, 1H), 7.22–7.15 (m, 2H), 7.01 (s, 1H), 6.93 (d, *J* = 8.0 Hz, 1H), 6.83 (dd, *J* = 8.2, 1.8 Hz, 1H), 5.53, 5.47 (s, 1H, NCHP), 4.30–4.22 (m, 2H, OCH_2_), 4.14–3.98 (m, 2H, OCH_2_), 1.33 (t, *J* = 7.2 Hz, 3H, CH_3_), 1.17 (t, *J* = 6.8 Hz, 3H, CH_3_). ^13^C-NMR (100 MHz, CDCl_3_): δ 162.98, 146.44, 139.07, 137.73, 131.78, 131.46, 130.95, 129.82, 128.23, 127.77, 123.13, 119.94, 116.16, 115.90, 114.91, 110.56, 63.94, 63.67, 48.68 (47.18), 16.46, 16.26. Anal. calcd. for C_21_H_22_F_3_N_2_O_4_P: C, 55.51; H, 4.88; N, 6.17. Found C, 55.48; H, 4.86; N, 6.19.

*Diethyl((2-oxo-1,2-dihydroquinolin-3-yl)((4-(trifluoromethyl)phenyl)amino)methyl)phosphonate* (**4f**). Following the general procedure the compound **4f** was obtained as a yellow solid, yield 78.0%, m.p. 219–222 °C. ^1^H-NMR (400 MHz, DMSO-*d*_6_): δ 12.03 (s, 1H, NH), 8.09 (d, *J* = 3.6 Hz, 1H), 7.61 (d, *J* = 7.8 Hz, 1H), 7.54–7.49 (m, 2H), 7.39 (d, *J* = 8.8 Hz, 1H), 7.33 (d, *J* = 8.1 Hz, 1H), 7.19 (t, *J* = 7.4 Hz, 1H), 7.13(t, 1H, *J* = 8 Hz, 1H), 6.84 (d, *J* = 8.7 Hz, 1H). 5.40–5.32 (m, 1H, NCHP), 4.12–3.90 (m, 4H, 2 × OCH_2_), 1.21 (t, 3H, *J* = 7.0 Hz, CH_3_), 1.10 (t, 3H, *J* = 7.0 Hz, CH_3_). ^13^C-NMR (100 MHz, DMSO): δ 161.37, 142.41, 138.06, 133.61, 130.54, 128.75, 127.75, 126.22, 122.60, 122.16, 118.77, 118.10, 115.10, 112.53, 62.87, 62.61, 47.25 (45.71), 16.22, 16.05. Anal. calcd. for C_21_H_22_F_3_N_2_O_4_P: C, 55.51; H, 4.88; N, 6.17. Found C, 55.53; H, 4.89; N, 6.18.

*Diethyl(((2-nitrophenyl)amino)(2-oxo-1,2-dihydroquinolin-3-yl)methyl)phosphonate* (**4g**). Following the general procedure the compound **4g** was obtained as a yellow solid, yield 75.6%, m.p. 207–209 °C. ^1^H-NMR (400 MHz, CDCl_3_): δ 11.27 (s, 1H, NH), 9.05(t, *J* = 8.9 Hz, 1H, NH), 8.20 (d, *J* = 7.3 Hz, 1H), 8.03 (d, *J* = 3.6 Hz, 1H), 7.59 (d, *J* = 7.9 Hz, 1H), 7.53 (t, *J* = 7.7 Hz, 1H), 7.36 (t, *J* = 7.2 Hz, 2H), 7.24 (t, *J* = 7.6 Hz, 1H), 6.87 (d, *J* = 8.5 Hz, 1H), 6.71 (t, *J* = 7.8 Hz, 1H), 5.74, 5.68 (s, 1H, NCHP), 4.29–4.16 (m, 2H, OCH_2_), 4.22–4.14 (m, 2H, OCH_2_), 1.36–1.28 (m, 6H, 2×CH_3_). ^13^C-NMR (100 MHz, CDCl_3_): δ 162.98, 143.61, 139.00, 137.87, 136.47, 133.40, 131.19, 128.29, 127.21, 126.90, 123.32, 119.96, 116.98, 116.10, 114.54, 64.08, 63.73, 48.60 (47.06), 16.47, 16.41. Anal. calcd. for C_20_H_22_N_3_O_6_P: C, 55.69; H, 5.14; N, 9.74. Found C, 55.67; H, 5.12; N, 9.71.

*Diethyl(((4-nitrophenyl)amino)(2-oxo-1,2-dihydroquinolin-3-yl)methyl)phosphonate* (**4h**). Following the general procedure the compound **4h** was obtained as a yellow solid, yield 81.4%, m.p. 221–224 °C. ^1^H-NMR (400 MHz, DMSO-*d*_6_): δ 12.11 (s, 1H, NH), 8.12(d, *J* = 3.2 Hz, 1H,), 8.02 (d, *J* = 9.2Hz, 2H), 7.96–7.92 (m, 1H), 7.65 (d, *J* = 7.6 Hz, 1H), 7.53 (t, *J* = 8.0 Hz, 1H), 7.20 (t, *J* = 7.6 Hz, 1H), 6.85 (d, *J* = 8.8 Hz, 2H), 5.48, 5.42 (d, *J* = 9.4 Hz, 1H, NCHP), 4.14–4.09 (m, 2H, OCH_2_), 4.04–3.93 (m, 2H, OCH_2_), 1.22 (t, *J* = 7.2 Hz, 3H, CH_3_), 1.11 (t, *J* = 7.2 Hz, 3H, CH_3_). ^13^C-NMR (100 MHz, DMSO-*d*_6_): δ 160.93, 153.18, 138.13, 138.01, 137.21, 130.69, 128.09, 127.85, 125.89, 122.20, 118.72, 118.69, 115.16, 111.94, 62.97, 62.87, 47.31 (45.75), 16.23, 16.06. Anal. calcd. for C_20_H_22_N_3_O_6_P: C, 55.69; H, 5.14; N, 9.74. Found C, 56.01; H, 5.11; N, 9.72.

*Diethyl(((4-methoxyphenyl)amino)(2-oxo-1,2-dihydroquinolin-3-yl)methyl)phosphonate* (**4i**). Following the general procedure the compound **4i** was obtained as a light yellow solid, yield 67.5%, m.p. 187–189 °C. ^1^H-NMR (400 MHz, CDCl_3_): δ 11.19 (s, 1H, NH), 8.03 (d, *J* = 3.7 Hz, 1H), 7.53 (d, *J* = 7.9 Hz, 1H), 7.48 (t, *J* = 7.7 Hz, 1H), 7.33 (d, *J* = 8.2 Hz, 1H), 7.19 (t, *J* = 7.6 Hz, 1H), 6.73–6.64 (m, 4H), 5.49, 5.43 (s, 1H, NCHP), 4.32–4.23 (m, 2H, OCH_2_), 4.12–3.97 (m, 2H, OCH_2_), 3.66 (s, 3H, CH_3_), 1.34 (t, *J* = 7.1 Hz, 3H, CH_3_), 1.15 (t, *J* = 7.0 Hz, 3H, CH_3_). ^13^C-NMR (100 MHz, DMSO-*d*_6_): δ 161.19, 151.69, 140.86, 137.98, 137.15, 130.28, 129.66, 127.61, 122.04, 118.92, 115.00, 114.45, 62.73, 62.41, 55.13, 48.45 (46.89), 16.28, 16.09. Anal. calcd. for C_21_H_25_N_2_O_5_P: C, 60.57; H, 6.05; N, 6.73. Found C, 60.53; H, 6.02; N, 6.70.

*Diethyl(((2-chlorophenyl)amino)(2-oxo-1,2-dihydroquinolin-3-yl)methyl)phosphonate* (**4j**)*.* Following the general procedure the compound **4j** was obtained as an orange solid, yield 91.7%, m.p. 174–177 °C. ^1^H-NMR (400 MHz, CDCl_3_) δ: 11.68 (s, 1H), 8.04 (d, *J* = 3.6 Hz, 1H), 7.58 (d, *J* = 7.9 Hz, 1H), 7.49 (t, *J* = 7.6 Hz, 1H), 7.37 (d, *J* = 8.2 Hz, 1H), 7.21 (t, *J* = 7.5 Hz, 1H), 7.02 (d, *J* = 8.4 Hz, 2H), 6.66 (d, *J* = 8.8 Hz, 2H), 5.55, 5.49 (s, 1H, NCHP), 4.31–4.25 (m, 2H, OCH_2_), 4.16–4.03 (m, 2H, OCH_2_), 1.35 (t, *J* = 7.1 Hz, 3H, CH_3_), 1.15 (t, *J* = 7.1 Hz, 3H, CH_3_). ^13^C-NMR (100 MHz, CDCl_3_): δ 163.30, 142.03, 138.67, 137.84, 130.81, 129.30, 128.20, 127.95, 127.75, 123.02, 120.01, 119.93, 118.75, 115.96, 112.44, 64.02, 63.63, 49.03 (47.49), 16.51, 16.33. Anal. calcd. for C_20_H_22_ClN_2_O_4_P: C, 57.08; H, 5.27; N, 6.66. Found C, 57.06; H, 5.25; N, 6.69.

*Diethyl(((3-chlorophenyl)amino)(2-oxo-1,2-dihydroquinolin-3-yl)methyl)phosphonate* (**4k**). Following the general procedure the compound **4k** was obtained as a yellow solid, yield 89.3%, m.p. 170–172 °C. ^1^H-NMR (400 MHz, CDCl_3_): δ 12.02 (s, 1H), 8.08 (d, *J* = 3.6 Hz, 1H), 7.53 (d, *J* = 7.9 Hz, 1H), 7.48 (t, *J* = 7.7 Hz, 1H), 7.39 (d, *J* = 8.2 Hz, 1H), 7.20 (t, *J* = 7.5 Hz, 1H), 7.01 (t, *J* = 8.1 Hz, 1H), 6.75 (t, *J* = 2.0 Hz, 1H), 6.66 (dd, *J* = 7.7, 1.4 Hz, 1H), 6.58 (dd, *J* = 8.2, 2.1 Hz, 1H), 5.50–5.44 (m, 1H, NCHP), 4.32–4.23 (m, 2H, OCH_2_), 4.10–3.7(m, 2H, OCH_2_), 1.34 (t, *J* = 7.1 Hz, 3H, CH_3_), 1.15 (t, *J* = 7.1 Hz, 3H, CH_3_). ^13^C-NMR (100 MHz, CDCl_3_): δ 163.28, 147.69, 139.02, 137.98, 134.98, 130.73, 130.29, 128.16, 128.08, 122.84, 119.92, 118.31, 115.92, 113.86, 111.69, 64.05, 63.72, 48.57 (47.03), 16.52, 16.29. Anal. calcd. for C_20_H_22_ClN_2_O_4_P: C, 57.08; H, 5.27; N, 6.66. Found C, 57.10; H, 5.26; N, 6.68.

*Diethyl(((4-chlorophenyl)amino)(2-oxo-1,2-dihydroquinolin-3-yl)methyl)phosphonate* (**4l**). Following the general procedure the compound **4l** was obtained as a yellow solid, yield 88.1%, m.p. 192–194 °C. ^1^H-NMR (400 MHz, CDCl_3_): δ 11.65 (s, 1H, NH), 8.06 (d, *J* = 3.6 Hz, 2H), 7.54–7.48 (m, 2H), 7.36 (d, *J* = 8.2 Hz, 1H), 7.21 (t, *J* = 7.3 Hz, 1H), 7.05 (d, *J* = 8.8 Hz, 1H), 6.66 (d, *J* = 8.8 Hz, 2H), 5.51, 5.45 (s, 1H, NCHP), 4.34–4.21 (m, 2H, OCH_2_), 4.10–3.99 (m, 2H, OCH_2_), 1.35 (t, *J* = 7.1 Hz, 3H, CH_3_), 1.15 (t, *J* = 7.1 Hz, 3H, CH_3_). ^13^C-NMR (100 MHz, CDCl_3_): δ 163.21, 144.83, 138.81, 137.94, 130.76, 129.12, 128.19, 128.13, 123.20, 122.90, 119.89, 115.80, 114.97, 63.95, 63.59, 48.90 (47.35), 16.52, 16.29. Anal. calcd. for C_20_H_22_ClN_2_O_4_P: C, 57.08; H, 5.27; N, 6.66. Found C, 57.05; H, 5.29; N, 6.64.

*Diethyl(((2-bromophenyl)amino)(2-oxo-1,2-dihydroquinolin-3-yl)methyl)phosphonate* (**4m**). Following the general procedure the compound **4m** was obtained as a yellow solid, yield 84.8%, m.p. 209–211 °C. ^1^H-NMR (400 MHz, CDCl_3_): δ 11.62 (s, 1H, NH), 8.02 (d, *J* = 3.6 Hz, 1H), 7.58 (d, *J* = 8.0 Hz, 1H), 7.46 (t, *J* = 8.0 Hz, 1H), 7.43–7.38 (m, 2H), 7.22 (t, *J* = 3.6 Hz, 1H), 7.05 (t, *J* = 7.6 Hz, 1H), 6.65 (d, *J* = 8.0 Hz, 1H), 6.58 (t, *J* = 7.6 Hz, 1H), 5.53–5.47 (m, 1H, NCHP), 4.33–4.26 (m, 2H, OCH_2_), 4.14–4.06 (m, 2H, OCH_2_), 1.35 (t, *J* = 6.8Hz, 3H, CH_3_), 1.20 (t, *J* = 7.2 Hz, 3H, CH_3_). ^13^C-NMR (100 MHz, CDCl_3_): δ 163.44, 142.94, 138.51, 138.00, 132.54, 130.72, 128.61, 128.24, 127.94, 122.89, 119.94, 119.21, 115.92, 112.52, 110.44, 63.95, 63.54, 49.23 (47.70), 16.56, 16.36. Anal. calcd. for C_20_H_22_BrN_2_O_4_P: C, 51.63; H, 4.77; N, 6.02. Found C, 51.65; H, 4.75; N, 6.04.

*Diethyl(((3-bromophenyl)amino)(2-oxo-1,2-dihydroquinolin-3-yl)methyl)phosphonate* (**4n**). Following the general procedure the compound **4n** was obtained as a light yellow solid, yield 63.0%, m.p. 213–216 °C. ^1^H-NMR (400 MHz, CDCl_3_): δ 12.14 (s, 1H, NH), 8.10 (d, *J* = 3.6 Hz, 1H), 7.52 (d, *J* = 8.0 Hz, 1H), 7.45 (t, *J* = 8.0 Hz, 1H), 7.38 (d, *J* = 8.0 Hz, 1H), 7.18 (t, *J* = 8.0 Hz, 1H), 6.97–6.92 (m, 2H), 6.80 (dd, *J* = 2.0, 1.2 Hz,1H), 6.64 (dd, *J* = 1.6, 2.4 Hz, 1H), 5.51, 5.45 (s, 1H, NCHP), 4.31–4.23 (m, 2H, OCH_2_), 4.13–3.97 (m, 2H, OCH_2_), 1.35 (t, *J* = 5.6 Hz, 3H, CH_3_), 1.17 (t, *J* = 3.2 Hz, 3H, CH_3_). ^13^C-NMR (100 MHz, CDCl_3_): δ 163.17, 147.69, 139.06, 137.88, 130.81, 130.60, 128.15, 127.89, 123.21, 122.94, 121.30, 119.90, 116.80, 115.99, 112.07, 64.02, 63.70, 48.68 (47.13), 16.52, 16.30. Anal. calcd. for C_20_H_22_BrN_2_O_4_P: C, 51.63; H, 4.77; N, 6.02. Found C, 51.61; H, 4.78; N, 6.01.

*Diethyl(((4-bromophenyl)amino)(2-oxo-1,2-dihydroquinolin-3-yl)methyl)phosphonate* (**4o**). Following the general procedure the compound **4o** was obtained as a light yellow solid, yield 55.4%, m.p. 150–153 °C. ^1^H-NMR (400 MHz, CDCl_3_): δ 12.36 (s, 1H, NH), 8.10 (d, *J* = 3.6 Hz, 1H), 7.48 (t, *J* = 9.1 Hz, 2H), 7.40 (d, *J* = 8.1 Hz, 1H), 7.21–7.14 (m, 3H), 6.65 (d, *J* = 8.8 Hz, 2H), 5.56, 5.49 (s, 1H, NCHP), 4.32–4.24 (m, 2H, OCH_2_) 4.13–3.96 (m, 2H, OCH_2_), 1.34 (t, *J* = 7.1 Hz, 3H), 1.14 (t, *J* = 7.0 Hz, 3H, CH_3_). ^13^C-NMR (100 MHz, CDCl_3_): δ 158.12, 140.22, 140.07, 133.85, 132.84, 127.11, 125.93, 123.30, 123.03, 118.12, 114.96, 110.85, 110.44, 105.33, 59.03, 58.69, 43.94, 42.32, 11.61, 11.38. Anal. calcd. for C_20_H_22_BrN_2_O_4_P: C, 51.63; H, 4.77; N, 6.02. Found C, 51.66; H, 4.74; N, 6.05.

*Diethyl(((2,6-dimethylphenyl)amino)(2-oxo-1,2-dihydroquinolin-3-yl)methyl)phosphonate* (**4p**). Following the general procedure the compound **4p** was obtained as a brown solid, yield 68.7%, m.p. 213–216 °C. ^1^H-NMR (400 MHz, CDCl_3_): δ 11.46 (s, 1H, NH), 8.11 (d, *J* = 3.0 Hz, 1H), 7.61 (d, *J* = 7.9 Hz, 1H), 7.50 (t, *J* = 7.7 Hz, 1H), 7.34 (d, *J* = 8.2 Hz, 1H), 7.23 (d, *J* = 7.7 Hz, 1H), 6.93 (d, *J* = 7.5 Hz, 2H), 6.76 (t, *J* = 7.4 Hz, 1H), 5.25, 5.19 (s, 1H, NCHP), 4.18–4.11 (m, 2H, OCH_2_), 4.05–3.86 (m, 2H, OCH_2_), 2.33 (s, 6H, 2× CH_3_), 1.24 (t, 3H, *J* = 6.9 Hz, CH_3_), 1.10 (t, 3H, *J* = 7.0 Hz, CH_3_). ^13^C-NMR (100 MHz, CDCl_3_): δ 163.18, 144.57, 139.09, 137.93, 130.52, 129.94, 129.05, 128.55, 128.01, 122.85, 121.75, 119.93, 115.98, 63.16, 63.11, 52.38 (51.39), 18.84, 16.32, 16.26. Anal. calcd. for C_22_H_27_N_2_O_4_P: C, 63.76; H, 6.57; N, 6.76. Found C, 63.72; H, 6.59; N, 6.75.

*Diethyl(((3,4-dimethylphenyl)amino)(2-oxo-1,2-dihydroquinolin-3-yl)methyl)phosphonate* (**4q**). Following the general procedure the compound **4q** was obtained as a yellow solid, yield 91.5%, m.p. 185–188 °C. ^1^H-NMR (400 MHz, CDCl_3_): δ 12.06 (s, 1H, NH), 8.06 (d, *J* = 4.0 Hz, 1H), 7.51 (t, *J* = 7.6 Hz, 1H), 7.46 (d, *J* = 7.2 Hz, 1H), 7.40 (d, *J* = 8.4 Hz, 1H), 7.17 (t, *J* = 7.6 Hz, 1H), 6.85 (d, *J* = 8.0 Hz,1H), 6.57 (d, *J* = 1.6 Hz, 1H), 6.49(dd, *J* = 2.4, 2.0 Hz,1H), 5.57, 5.51 (s, 1H, NCHP), 4.32–4.25 (m, 2H, OCH_2_), 4.10–3.98 (m, 2H, OCH_2_), 2.1 (s, 3H, CH_3_), 2.07 (s, 3H, CH_3_), 1.34 (t, *J* = 7.2 Hz, 3H, CH_3_), 1.12 (t, *J* = 7.2 Hz, 3H, CH_3_). ^13^C-NMR (100 MHz, CDCl_3_): δ 163.44, 144.22, 138.59, 137.99, 137.39, 130.45, 130.34, 128.81, 128.15, 126.52, 122.65, 120.01, 115.84, 115.72, 110.88, 63.92, 63.46, 48.99 (47.45), 20.00, 18.67, 16.55, 16.30. Anal. calcd. for C_22_H_27_N_2_O_4_P: C, 63.76; H, 6.57; N, 6.76. Found C, 63.78; H, 6.55; N, 6.78.

*Diethyl(((3,5-bis(trifluoromethyl)phenyl)amino)(2-oxo-1,2-dihydroquinolin-3-yl)methyl) phosphonate* (**4r**). Following the general procedure the compound **4r** was obtained as a white solid, yield 84.6%, m.p. 246–248 °C. ^1^H-NMR (400 MHz, DMSO-*d*_6_): δ 12.16 (s, 1H), 8.11 (d, *J* = 3.3 Hz, 1H), 7.65 (d, *J* = 7.8 Hz, 1H), 7.56–7.49 (m, 2H), 7.32 (d, *J* = 5.2 Hz, 3H), 7.20 (t, *J* = 7.6 Hz, 1H), 7.15 (s, 1H), 5.42–7.53 (m, 1H, NCHP), 4.13 (m, 2H, OCH_2_), 4.04–3.90 (m, 2H, OCH_2_), 1.22 (t, *J* = 7.0 Hz, 3H, CH_3_), 1.10 (t, *J* = 7.0 Hz, 3H, CH_3_). ^13^C-NMR (100 MHz, DMSO-*d*_6_): δ 161.36, 148.92, 138.41, 138.27, 131.38, 131.07, 128.42, 128.25, 125.13, 122.56, 119.02, 115.44, 112.80, 109.35, 63.20, 63.12, 47.40 (45.85), 16.52, 16.37. Anal. calcd. for C_22_H_21_F_6_N_2_O_4_P: C, 50.58; H, 4.05; N, 5.36. Found C, 50.56; H, 4.03; N, 5.39.

*Diethyl(((2,4-dinitrophenyl)amino)(2-oxo-1,2-dihydroquinolin-3-yl)methyl)phosphonate* (**4s**). Following the general procedure the compound **4s** was obtained as a red solid, yield 57.4%, m.p. 201–204 °C. ^1^H-NMR (400 MHz, DMSO-*d*_6_): δ 12.20 (s, 1H, NH), 9.70 (s, 1H), 8.89 (d, *J* = 2.8 Hz, 1H, NH), 8.34 (dd, *J* = 9.5, 2.4 Hz, 1H), 8.14 (d, *J* = 3.2 Hz, 1H), 7.68 (t, *J* = 7.8 Hz, 1H), 7.54 (t, *J* = 7.7 Hz, 1H), 7.35 (d, *J* = 8.1 Hz, 2H), 7.20 (t, *J* = 7.3 Hz, 1H), 7.11 (d, *J* = 9.6 Hz, 1H), 5.77, 5.71 (d, *J* = 8.3 Hz, 1H, NCHP), 4.21–4.11 (m, 2H, OCH_2_), 4.08–3.98 (m, 2H, OCH_2_), 1.25 (t, *J* = 7.0 Hz, 3H, CH_3_), 1.14 (t, *J* = 7.1 Hz, 3H, CH_3_). ^13^C-NMR (100 MHz, DMSO-*d*_6_): δ 160.94, 146.67, 138.44, 138.22, 136.10, 131.11, 130.92, 130.36, 128.16, 125.72, 123.34, 122.29, 118.68, 115.51, 115.13, 63.38, 63.32, 50.54 (49.02), 16.18, 16.09. Anal. calcd. for C_20_H_21_N_4_O_8_P: C, 50.43; H, 4.44; N, 11.76. Found C, 50.46; H, 4.41; N, 11.78.

*Diethyl(((2-chloro-4-nitrophenyl)amino)(2-oxo-1,2-dihydroquinolin-3-yl)methyl) phosphonate* (**4t**). Following the general procedure the compound **4t** was obtained as an orange solid, yield 70.0%, m.p. 137–139 °C. ^1^H-NMR (400 MHz, DMSO-*d*_6_): δ 12.15 (s, 1H), 8.23 (d, *J* = 2.6 Hz, 1H), 8.18 (d, *J* = 3.2 Hz, 1H), 8.10 (dd, *J* = 9.1, 2.8 Hz, 1H), 7.71 (d, *J* = 7.7 Hz, 1H), 7.54 (t, *J* = 7.7 Hz, 1H), 7.36 (d, *J* = 8.2 Hz, 1H), 7.22 (t, *J* = 7.5 Hz, 1H), 6.96 (d, *J* = 9.3 Hz, 1H), 5.63, 5.55 (d, *J* = 9.0 Hz, 1H, NCHP), 4.16–4.07 (m, 2H, OCH_2_), 4.05–3.96 (m, 2H, OCH_2_), 1.22 (t, *J* = 7.0 Hz, 3H, CH_3_), 1.12 (t, *J* = 7.0 Hz, 3H, CH_3_). ^13^C-NMR (100 MHz, DMSO-*d*_6_): δ 161.29, 147.65, 138.77, 138.12, 137.35, 130.84, 128.07, 125.57, 125.04, 124.75, 122.33, 117.80, 115.15, 113.52, 110.90, 63.08, 63.02, 50.99 (49.46), 16.20, 16.11. Anal. calcd. for C_20_H_21_ClN_3_O_6_P: C, 51.57; H, 4.54; N, 9.02. Found C, 51.55; H, 4.51; N, 9.05.

*Diethyl(((3-chloro-4-fluorophenyl)amino)(2-oxo-1,2-dihydroquinolin-3-yl)methyl)phosphonate* (**4u**). Following the general procedure the compound **4u** was obtained as a yellow solid, yield 85.2%, m.p. 157–159 °C. ^1^H-NMR (400 MHz, CDCl_3_): δ 11.51 (s, 1H), 8.06 (d, *J* = 3.6 Hz, 1H), 7.52 (dd, *J* = 15.2, 7.8 Hz, 2H), 7.36 (d, *J* = 8.3 Hz, 1H), 7.21 (t, *J* = 7.2 Hz, 1H), 6.87 (t, *J* = 8.8 Hz, 1H), 6.79 (dd, *J* = 6.0, 2.8 Hz, 1H), 6.58–6.54 (m, 1H), 5.46, 5.39 (s, 1H, NCHP), 4.32–4.23 (m, 2H, OCH_2_), 4.11–3.96 (m, 2H, OCH_2_), 1.34 (t, *J* = 7.1 Hz, 3H, CH_3_), 1.15 (t, *J* = 7.0 Hz, 3H, CH_3_). ^13^C-NMR (100 MHz, CDCl_3_): δ 163.20, 150.41, 143.03, 139.15, 137.94, 130.90, 128.19, 127.82, 122.99, 121.08, 119.93, 116.70, 115.97, 115.46, 112.93, 63.91, 63.58, 49.17, 47.62, 16.47, 16.24. Anal. calcd. for C_20_H_21_ClFN_2_O_4_P: C, 54.74; H, 4.82; N, 6.38. Found C, 54.71; H, 4.81; N, 6.40.

*Diethyl(((2,3-dichlorophenyl)amino)(2-oxo-1,2-dihydroquinolin-3-yl)methyl)phosphonate* (**4v**). Following the general procedure the compound **4v** was obtained as a light yellow solid, yield 70.3%, m.p. 172–175 °C. ^1^H-NMR (400 MHz, CDCl_3_) δ: 11.77 (s, 1H), 8.02 (d, *J* = 3.6 Hz, 1H), 7.59 (d, *J* = 7.8 Hz, 1H), 7.52 (t, *J* = 7.7 Hz, 1H), 7.40 (d, *J* = 8.1 Hz, 1H), 7.23 (t, *J* = 7.4 Hz, 1H), 6.93 (t, *J* = 8.1 Hz, 1H), 6.80 (d, *J* = 7.0 Hz, 1H), 6.59 (d, *J* = 7.7 Hz, 1H), 5.74 (t, *J* = 8.5 Hz, 1H, NH), 5.57, 5.51 (d, *J* = 7.8, 12.7 Hz, 1H, NCHP), 4.32–4.25 (m, 2H, OCH_2_), 4.16–4.03 (m, 2H, OCH_2_), 1.35 (t, *J* = 7.0 Hz, 3H, CH_3_), 1.19 (t, *J* = 7.0 Hz, 3H, CH_3_). ^13^C-NMR (100 MHz, CDCl_3_): δ 163.39, 143.66, 138.61, 138.00, 133.06, 130.86, 128.26, 127.85, 122.99, 119.84, 119.81, 119.38, 118.11, 115.88, 110.29, 63.99, 63.67, 49.32 (47.79), 16.53, 16.34. Anal. calcd. for C_20_H_21_Cl_2_N_2_O_4_P: C, 52.76; H, 4.65; N, 6.15. Found C, 52.79; H, 4.62; N, 6.11.

*Diethyl(((3,5-dichlorophenyl)amino)(2-oxo-1,2-dihydroquinolin-3-yl)methyl) phosphonate* (**4w**). Following the general procedure the compound **4w** was obtained as a white solid, yield 84.4%, m.p. 240–242 °C. ^1^H-NMR (400 MHz, DMSO-*d*_6_): δ 12.13 (s, 1H, NH), 8.07 (d, *J* = 3.2 Hz, 1H), 7.64 (d, *J* = 7.8 Hz, 1H), 7.53 (t, *J* = 7.7 Hz, 1H), 7.34 (d, *J* = 8.2 Hz, 1H), 7.22 (t, *J* = 7.4 Hz, 1H), 6.74 (s, 2H), 6.69 (s, 1H), 5.28, 5.22 (d, *J* = 9.5 Hz, 1H, NCHP), 4.18–4.09 (m, 2H, OCH_2_), 4.05–3.86 (m, 2H, OCH_2_), 1.23 (t, *J* = 7.0 Hz, 3H, CH_3_), 1.10 (t, *J* = 7.0 Hz, 3H, CH_3_). ^13^C-NMR (100 MHz, DMSO-*d*_6_): δ 161.31, 149.81, 138.35, 137.97, 134.64, 131.00, 128.81, 128.16, 122.57, 119.03, 116.24, 115.45, 111.53, 63.24, 63.07, 47.47 (45.92), 16.58, 16.39. Anal. calcd. for C_20_H_21_Cl_2_N_2_O_4_P: C, 52.76; H, 4.65; N, 6.15. Found C, 52.74; H, 4.67; N, 6.13.

*Diethyl((naphthalen-1-ylamino)(2-oxo-1,2-dihydroquinolin-3-yl)methyl)phosphonate* (**4x**). Following the general procedure the compound **4x** was obtained as a light yellow solid, yield 79.1%, m.p. 213–215 °C. ^1^H-NMR (400 MHz, CDCl_3_): δ 11.57 (s, 1H, NH), 8.09 (t, *J* = 5.6 Hz, 2H,), 7.79 (d, *J* = 8.5 Hz, 1H), 7.55–7.46 (m, 4H), 7.40 (d, *J* = 7.8 Hz, 1H), 7.23 (s, 1H), 7.22–7.16 (m, 2H), 6.61 (d, *J* = 7.2 Hz, 1H), 5.71, 5.65 (s, 1H, NCHP), 4.36–4.28 (m, 2H, OCH_2_), 4.17–4.02 (m, 2H, OCH_2_), 1.35 (t, *J* = 7.0 Hz, 3H, CH_3_), 1.17 (t, *J* = 7.0 Hz, 3H, CH_3_). ^13^C-NMR (100 MHz, CDCl_3_): δ 163.37, 140.65, 138.60, 137.85, 134.35, 130.78, 128.69, 128.22, 127.73, 126.45, 125.99, 125.27, 123.97, 123.03, 120.30, 120.01, 119.12, 115.98, 106.52, 63.95, 63.66, 49.66 (48.13), 16.54, 16.34. Anal. calcd. for C_24_H_25_N_2_O_4_P: C, 66.05; H, 5.77; N, 6.42. Found C, 66.07; H, 5.74; N, 6.45.

### 3.2. Biological Assays

#### 3.2.1. Cell Antiproliferative Activity Assay

The MTT method was used to evaluate the antiproliferative activities of the synthesized 2-oxoquinoline aminophosphonate derivatives **4a**–**x**. The A549, HeLa, MCF-7, and U2OS cells lines used in this study were all obtained from the Institute of Biochemistry and Cell Biology, China Academy of Sciences. All cells lines were seeded in 96-well plates at a density of 5 × 10^4^ cells/mL and then cultivated at 37 °C for 24 h. In order to investigate the potential of compounds **4a**–**x**, 5-FU, a commercial classical anticancer drug which was used as a reference organic drug, was added to each well (final concentrations: 0.5, 0.3, 0.1, 0.03, and 0.01 µM, respectively). After co-incubation for 48 h, 60 µL of MTT solution in phosphate buffered saline (PBS, 2.5 mg/mL) was added to each well, and the cells were incubated for another 4 h at 37 °C in a humid atmosphere of 5% CO_2_/95% air. After drawing-off of the culture medium, 150 mL of DMSO was added to dissolve formazan crystals; the percentage of cell viability was determined using a microplate reader. The absorbance was read by an enzyme labeling instrument with a 570 nm wavelength measurement. The cytotoxicity was estimated based on the percentage of cell survival in a dose dependent manner relative to the negative control. The final IC_50_ values were calculated by the Bliss method. All assays were conducted with four parallel samples.

#### 3.2.2. AO/EB Staining

HeLa cells were plated in a glass-bottom dish (glass diameter 10 mm) at 1.0 × 10^4^ cells/dish and cultured for 24 h at 37 °C in 5% humidified CO_2_. After the culture medium was removed, 1 mL of fresh medium, 10% fetal bovine serum, and compound **4u** (10 µM) were added to the cells. After incubation for 12 or 24 h at 37 °C in 5% CO_2_, then 2 mL of AO/EB (Dojindo Laboratories, Beijing, China) stain (20 µM) was added to the cells, and the cells were washed twice with PBS before they were observed by Confocal Laser Scanning Fluorescence Microscopy (CLSFM) (FV1000 (TY1318), Olympus, Japan). In the case of the compound (**4u**)-Acridine orange(AO)/ethidium bromide(EB) system, the cells were cultured for 20 min, and CLSFM observations were carried out with excitation by an argon laser (488 nm for AO) and Helium-Neon laser (543 nm for EB). The emitted fluorescence was detected through a 505–550 nm bandpass filter and a 610–690 nm long-pass filter, respectively.

#### 3.2.3. Hoechst 33342 Staining

HeLa cells were seeded in six-well plates at a density of 1 × 10^4^ cells/mL and then cultivated at 37 °C for 24 h. The cells were treated with compound **4u** (5 and 10 µM,) for 12 h. Next, the cells were washed twice with PBS and stained with 1 mL of Hoechst 33342 (Dojindo Laboratories) for 15 min, then washed twice with PBS. Subsequently, the stained nuclei were observed under a Nikon TE300 fluorescence microscope using 350 nm excitation and 460 nm emission.

#### 3.2.4. Apoptosis Detection via Flow Cytometry

The extent of apoptosis was quantitatively measured using an Annexin V binding assay [27]. HeLa cells were cultured in Minimum Essential Medium (MEM) supplemented with 10% heat-inactivated fetal bovine serum (FBS). Cells were plated in a six-well culture dish at a density of 2.0 × 10^4^ cells/well and cultured for 24 h at 37 °C in 5% CO_2_. After the medium was removed, 1 mL of fresh MEM containing the indicated concentrations of compound **4u** was added and the cells were cultured at 37 °C in 5% CO_2_. Then, the cells floating in the supernatant were combined with the adherent fraction and washed with PBS thrice. The cells were incubated with Annexin V-FITC (Dojindo Laboratories, Beijing, China) and PI for 15 min at 37 °C in the dark. The samples were immediately analyzed using a Kaluza flow cytometry Software (Becton Dickinson, San Jose, CA, USA). A total of 2 × 10^4^ cells were collected for each sample. All experiments were performed in triplicate.

#### 3.2.5. Cell Cycle Distribution Analysis

Cell cycle distributions in cells were determined through PI (Propidium Iodide) staining, as previously described [27]. In brief, after HeLa cells were treated with compound **4u**, the suspension and adherent portions were collected into flow cytometry tubes and centrifuged at 1000 rpm for 5 min to obtain cell pellets. The cells were washed with PBS (phosphate buffer saline) and then fixed with 70% ethanol (−20 °C) overnight. Fixed cells were washed with PBS and sequentially incubated with RNase A (0.1 mg/mL) for 30 min and PI (50 mg/mL) for 15 min in the dark. The distribution of the cell cycle was determined using flow cytometry. The data were analyzed using ModFit software (Becton Dickinson, San Jose, CA, USA). All assays were conducted with three parallel samples, with 1 × 10^4^ cells collected for each sample.

## 4. Conclusions

A convenient one-pot three component reaction has been developed for the synthesis of 2-oxoquinoline α-aminophosphonate derivatives employing 2-oxoquinoline, aromatic amine, and diethyl phosphate. A series of novel 2-oxoquinoline α-aminophosphonate derivatives **4a**–**x** were successfully synthesized and were tested their antiproliferative activities against four cancer cell lines (A549, HeLa, MCF-7, and U2OS) using MTT assay. The *in vitro* cytotoxicity screening revealed that compounds **4j**, **4q**, **4r**, **4s**, **4u**, and **4x** exhibited better inhibitory activities than the anticancer drug 5-FU on the A549 cell line, with IC_50_ in the range of 16.6 ± 0.9–31.1 ± 0.1 µM, and compounds **4b**–**f**, **4j**, **4l**, **4n**, **4r**–**v**, and **4x** showed more cytotoxic inhibition than 5-FU on the HeLa cell line, with IC_50_ in the range of 2.5 ± 0.6–50.0 ± 0.8 µM, while all compounds except compounds **4a**, **4c**, **4g**, **4o**, **4p**, and **4q** demonstrated better cytotoxic inhibition than 5-FU on the MCF-7 cell line, with IC_50_ in the range of 0.3 ± 0.1–65.5 ± 2.7 µM. In addition, compounds **4p**, **4u**, and **4x** showed more potent cytotoxic inhibition than 5-FU on the O2US cell line, with IC_50_ in the range of 2.5 ± 0.2–26.3 ± 1.3 µM. The apoptosis-inducing activity of the representative compound **4u** in HeLa cells was investigated by AO/EB staining, Hoechst 33324 staining, and flow cytometry. *In vitro* pharmacological analysis demonstrated that compound **4u** exerted its antiproliferative activity against the HeLa cell line by inducing apoptosis, and arresting the cell cycle at the S and G2 phases. The above results indicated that the rational design of 2-oxoquinoline aminophosphonate derivatives as novel anticancer candidates is feasible. Further investigation of the mechanism of these compounds in the treatment of malignant tumors in humans is currently under way.

## Figures and Tables

**Table 1:** Effect of compounds **4a**–**x** against cell viability of different cell lines.

Compound	IC_50_ ± SD (µM) ^a^				
	Ar	A549	HeLa	MCF-7	U2OS
**4a**	Ph	103.6 ± 1.0	125.6 ± 4.4	90.2 ± 0.3	>200
**4b**	2-CH_3_C_6_H_4_	96.7 ± 5.7	25.5 ± 0.9	57.9 ± 0.3	143.1 ± 6.2
**4c**	3-CH_3_C_6_H_4_	86.7 ± 3.8	36.1 ± 2.6	112.2 ± 0.3	131.6 ± 5.3
**4d**	4-CH_3_C_6_H_4_	59.6 ± 4.3	49.4 ± 0.4	28.4 ± 0.4	>200
**4e**	3-CF_3_C_6_H_4_	91.7 ± 2.5	31.8 ± 0.6	29.1 ± 0.6	157.6 ± 4.1
**4f**	4-CF_3_C_6_H_4_	41.6 ± 2.1	39.7 ± 6.4	57.5 ± 1.4	58.5 ± 0.7
**4g**	2-NO_2_C_6_H_4_	53.5 ± 1.9	61.7 ± 0.9	174.3 ± 8.6	63.7 ± 1.2
**4h**	4-NO_2_C_6_H_4_	128.0 ± 1.2	67.9 ± 1.7	35.7 ± 0.8	73.0 ± 0.1
**4i**	4-OCH_3_C_6_H_4_	108.2 ± 1.1	73.4 ± 1.1	28.5 ± 1.9	109.9 ± 7.9
**4j**	2-ClC_6_H_4_	22.7 ± 3.0	50.0 ± 0.8	25.5 ± 0.4	73.9 ± 0.9
**4k**	3-ClC_6_H_4_	35.4 ± 0.6	137.0 ± 8.9	31.2 ± 2.0	60.8 ± 4.0
**4l**	4-ClC_6_H_4_	163.3 ± 3.3	49.9 ± 3.9	54.6 ± 1.4	110.5 ± 0.2
**4m**	2-BrC_6_H_4_	85.6 ± 2.2	95.2 ± 5.9	26.0 ± 0.5	86.5 ± 1.6
**4n**	3-BrC_6_H_4_	49.4 ± 2.5	19.2 ± 0.4	55.2 ± 2.1	46.3 ± 1.4
**4o**	4-BrC_6_H_4_	>200	>200	>200	94.9 ± 2.1
**4p**	2,6-diCH_3_C_6_H_3_	40.8 ± 0.4	64.9 ± 6.1	87.4 ± 3.3	26.3 ± 1.3
**4q**	3,4-diCH_3_C_6_H_3_	26.4 ± 1.4	76.8 ± 8.6	79.0 ± 1.9	89.8 ± 1.1
**4r**	3,5-diCF_3_C_6_H_3_	31.1 ± 0.1	45.9 ± 1.5	42.8 ± 5.9	132.1 ± 8.2
**4s**	2,4-diNO_2_C_6_H_3_	21.6 ± 1.2	37.2 ± 5.4	65.5 ± 2.7	87.1 ± 7.8
**4t**	2-Cl-4-NO_2_C_6_H_3_	62.7 ± 7.2	15.1 ± 1.4	63.8 ± 3.1	38.1 ± 1.5
**4u**	3-Cl-4-FC_6_H_3_	16.6 ± 0.9	2.5 ± 0.6	1.0 ± 0.4	26.3 ± 2.0
**4v**	2,3-diClC_6_H_3_	49.8 ± 7.8	39.3 ± 5.2	32.0 ± 1.4	44.7 ± 0.8
**4w**	3,5-diClC_6_H_3_	46.9 ± 0.8	62.2 ± 4.5	27.0 ± 3.4	43.4 ± 1.2
**4x**	1-Naphthyl	29.4 ± 0.5	27.4 ± 6.0	0.3 ± 0.1	2.5 ± 0.2
2-oxo-quinoline		>200	>200	>200	>200
5-FU		34.32 ± 2.1	56.5 ± 3.4	79.3 ± 5.1	NT ^b^

^a^ Results are expressed as means ± SD (standard deviation) of four independent experiments; ^b^ NT: not tested; 5-FU: 5-fluorouracil.

**Table 2:** Quantitative apoptosis assay of HeLa using Annexin V-FITC (Fluorescein Isothiocyanate)/PI (Propidium Iodide) dual staining method.

Group	Concentration (µM)	Necrotic Cells (Q1, %)	Late Apoptotic Cells (Q2, %)	Viable Cells (Q3, %)	Early Apoptotic Cells (Q4, %)	Apoptotic Cells (Q2 + Q4, %)
Control	0	0.06	0.2	99.23	0.51	0.71
Compd. **4u**	10	1.67	36.74	12.96	48.63	85.37
Compd. **4u**	15	1.96	32.17	7.42	58.46	90.63

**Table 3:** Effects of compound **4u** on cell cycle progression in HeLa cells.

Group	Concentration (µM)	G0/G1 (Phase%)	S (Phase%)	G2/M (Phase%)
Control	0	59.90	31.87	8.23
Compd. **4u**	5	44.18	41.56	14.27
Compd. **4u**	10	41.03	44.34	14.63

## Supplementary Materials

Supplementary materials can be found at http://www.mdpi.com/1422-0067/17/5/653/s1.
