# Supplementary material for: One-Pot Three-Component Synthesis of Novel Diethyl((2-oxo-1,2-dihydroquinolin-3-yl)(arylamino)methyl)phosphonate as Potential Anticancer Agents
Source: Int J Mol Sci. 2016 Apr 29;17(5):653. doi: 10.3390/ijms17050653 (PMC4881479; doi:10.3390/ijms17050653)
Supplement: Supplementary file 1 [file ijms-17-00653-s001.pdf]

Supplementary Materials: One-Pot Three-Component Synthesis of Novel diethyl ((2-oxo-1,2-dihydroquinolin-3-yl)(arylamino)methyl)phosphonate as Potential Anticancer Agents

Yi-Lin Fang, Zhi-Lin Wu, Meng-Wu Xiao, Yu-Ting Tang, Kang-Ming Li, Jiao Ye, Jian-Nan Xiang and Ai-Xi Hu

^{1}H-NMR and ^{13}C-NMR Spectrogram of compounds 4a–x

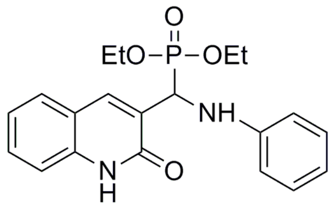

Figure S1. Chemical structure of compound **4a**.

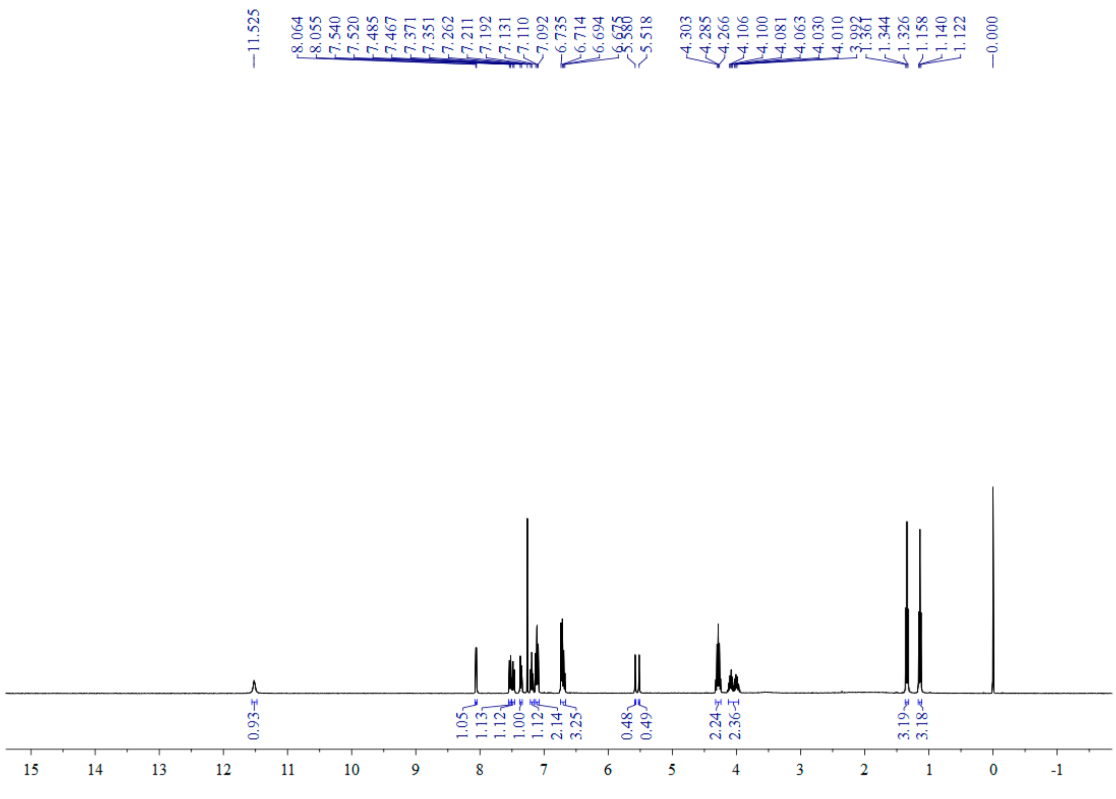

Figure S2. ^{1}H-NMR of compound **4a**.

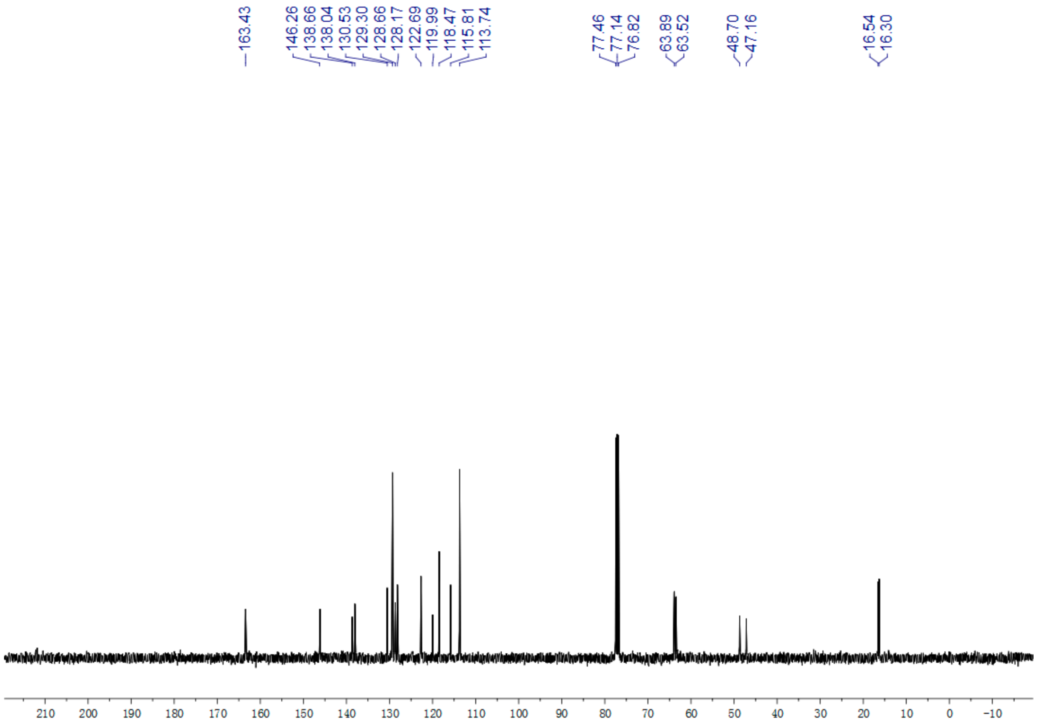

Figure S3. ^{13}C-NMR of compound **4a**.

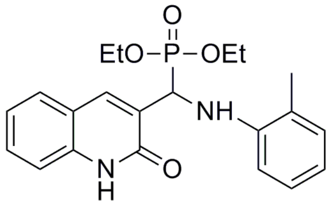

Figure S4. Chemical structure of compound **4b**.

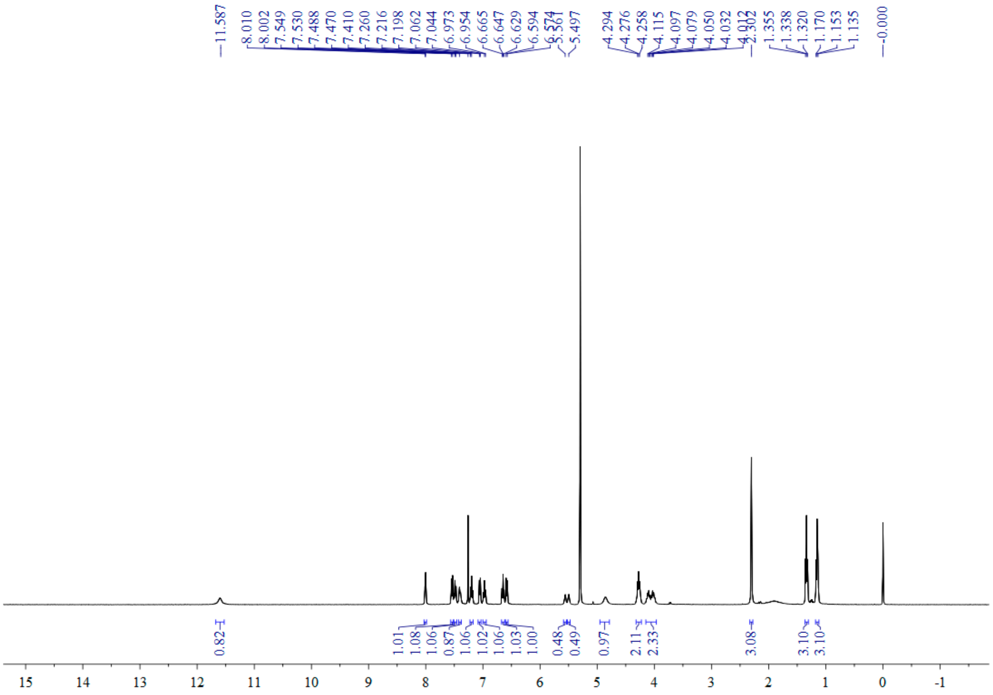

Figure S5. ^{1}H-NMR of compound **4b**.

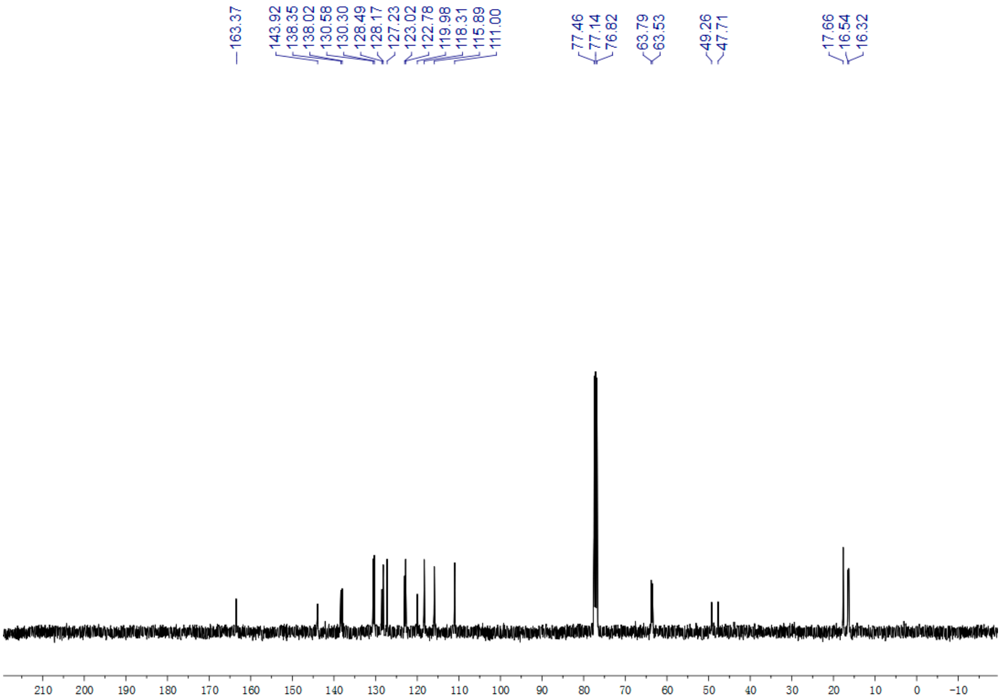

Figure S6. ^{13}C-NMR of compound **4b**.

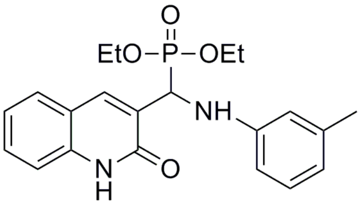

Figure S7. Chemical structure of compound **4c**.

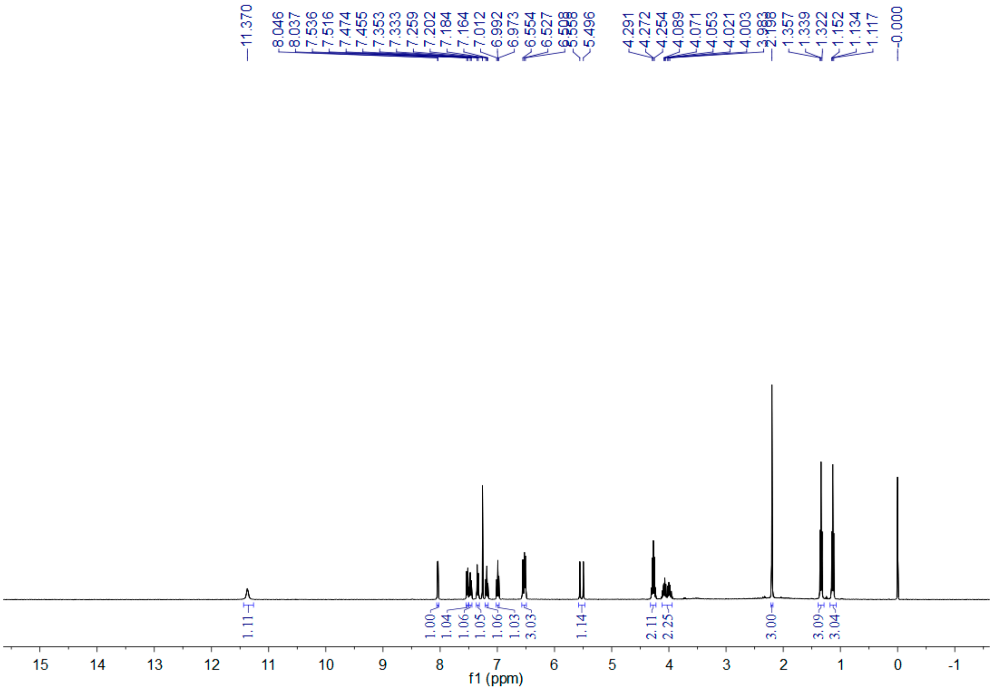

Figure S8. ^{1}H-NMR of compound **4c**.

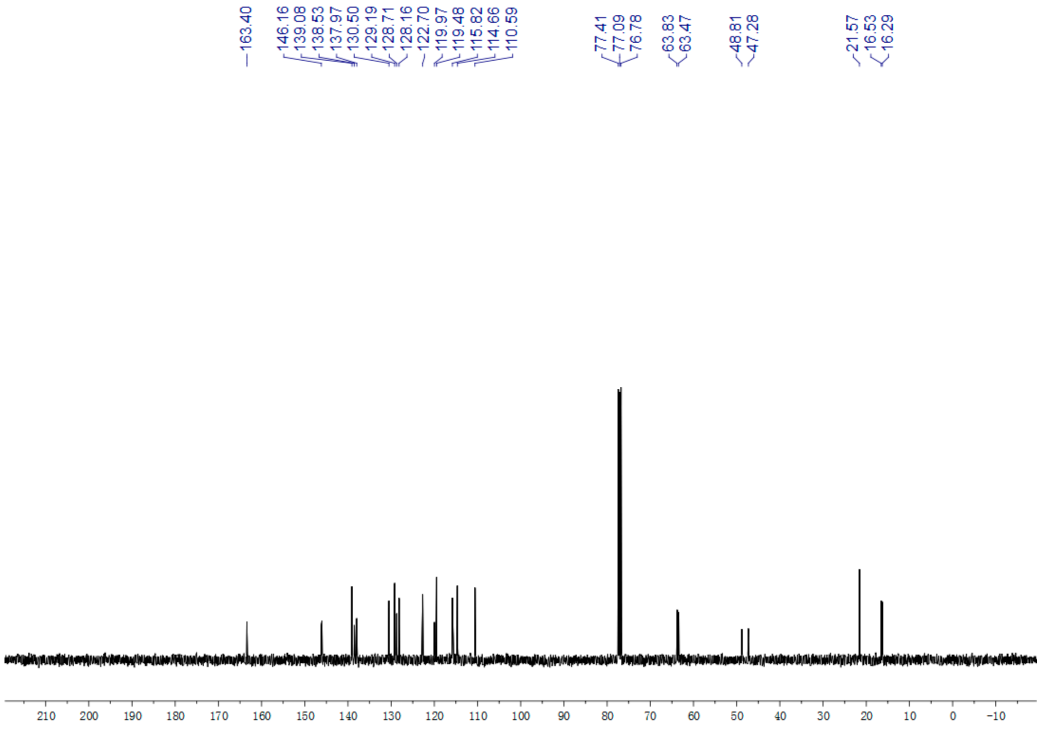

Figure S9. ^{13}C-NMR of compound **4c**.

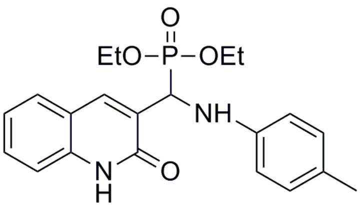

Figure S10. Chemical structure of compound **4d**.

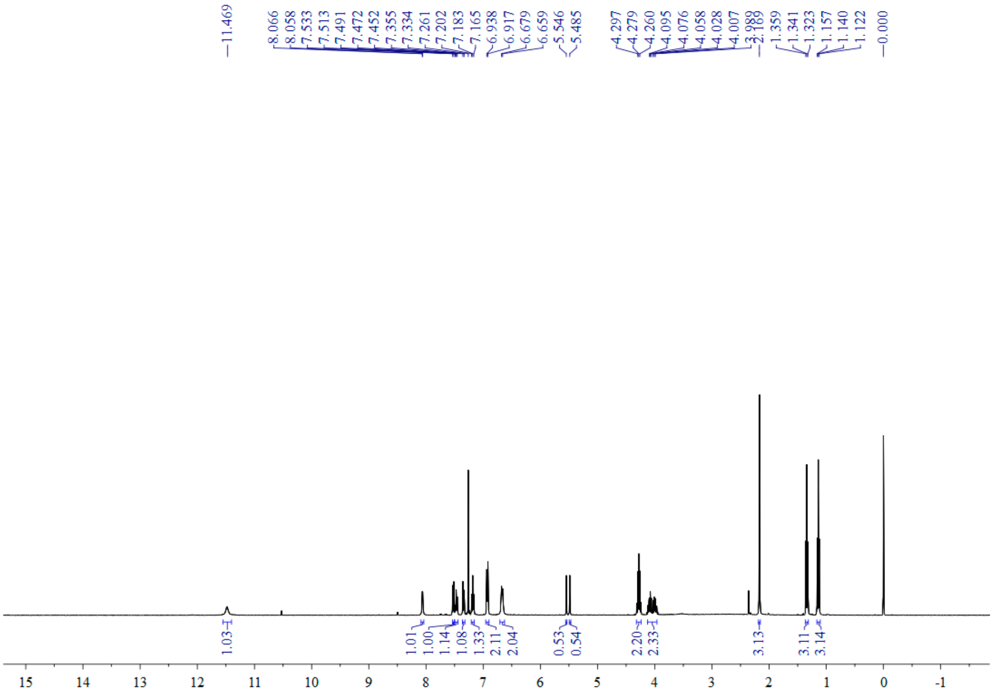

Figure S11. ^{1}H-NMR of compound **4d**.

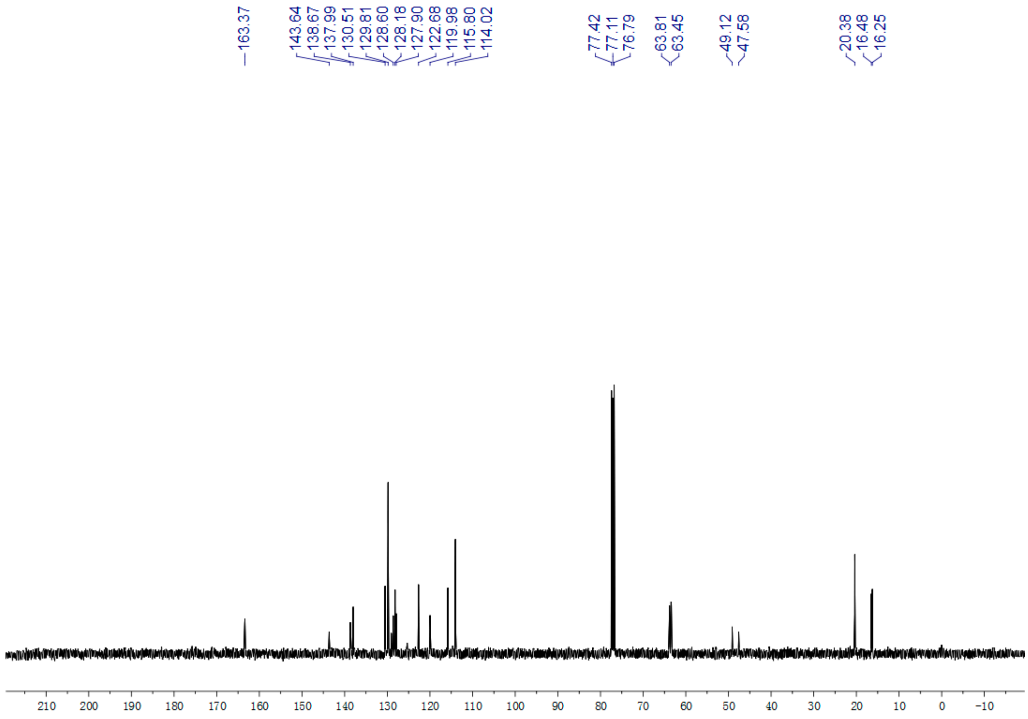

Figure S12. ^{13}C-NMR of compound **4d**.

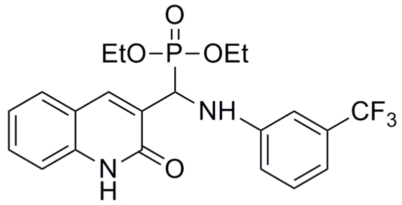

Figure S13. Chemical structure of compound **4e**.

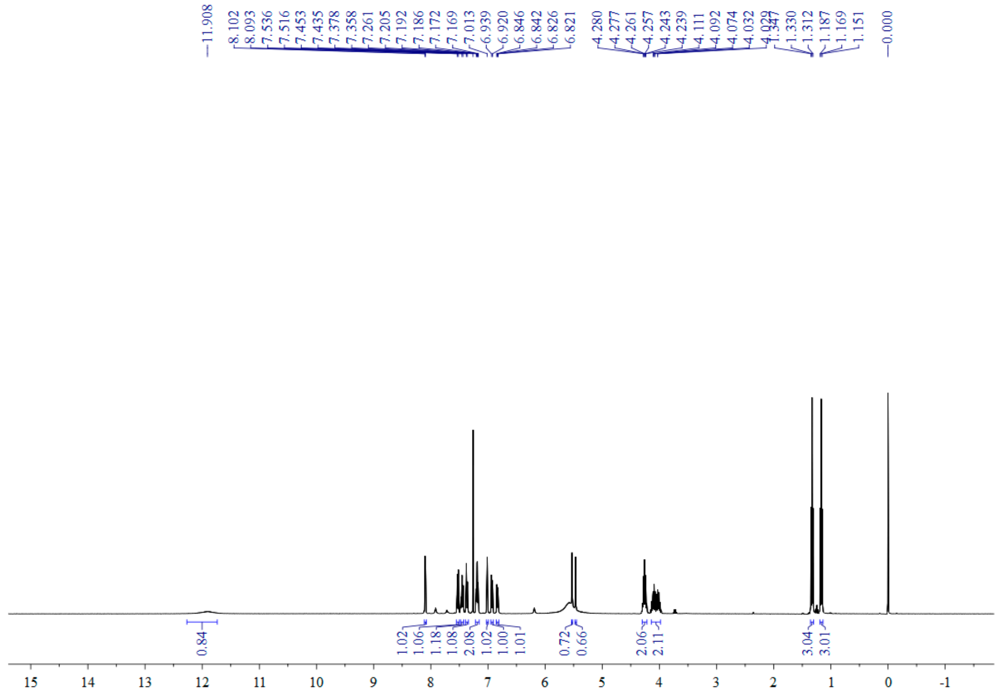

Figure S14. ^{1}H-NMR of compound **4e**.

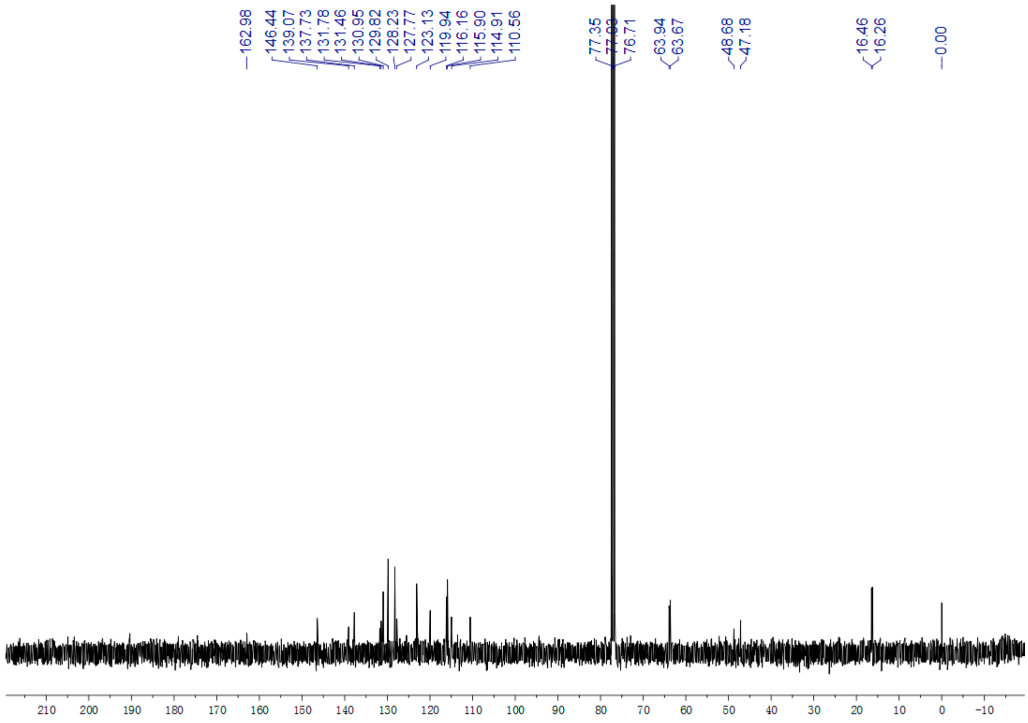

Figure S15. ^{13}C-NMR of compound **4e**.

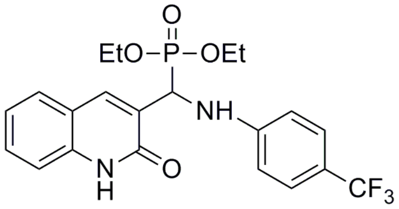

Figure S16. Chemical structure of compound **4f**.

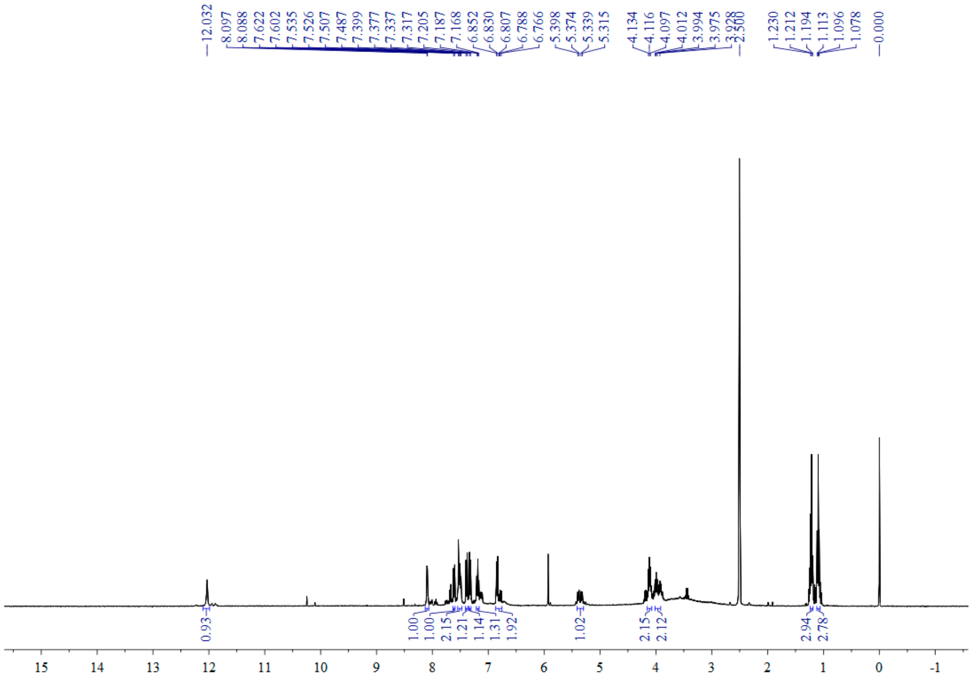

Figure S17. ^{1}H-NMR of compound **4f**.

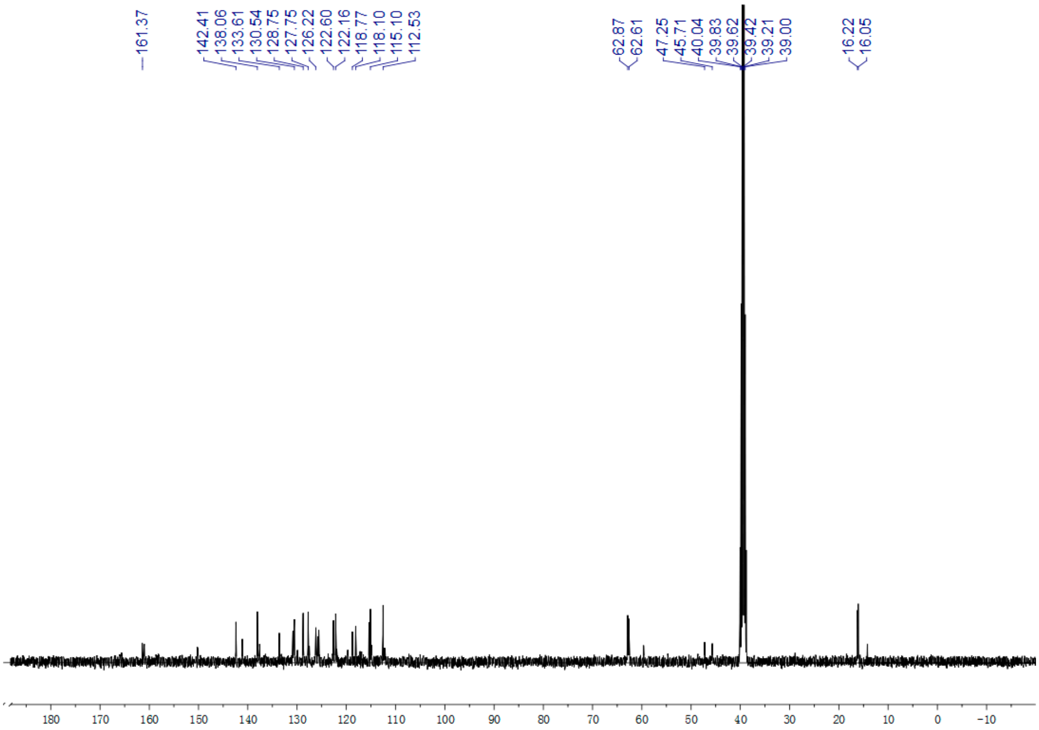

Figure S18. ^{13}C-NMR of compound **4f**.

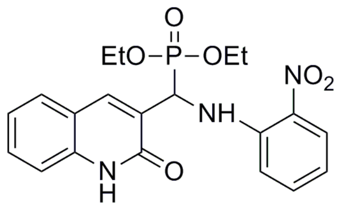

Figure S19. Chemical structure of compound **4g**.

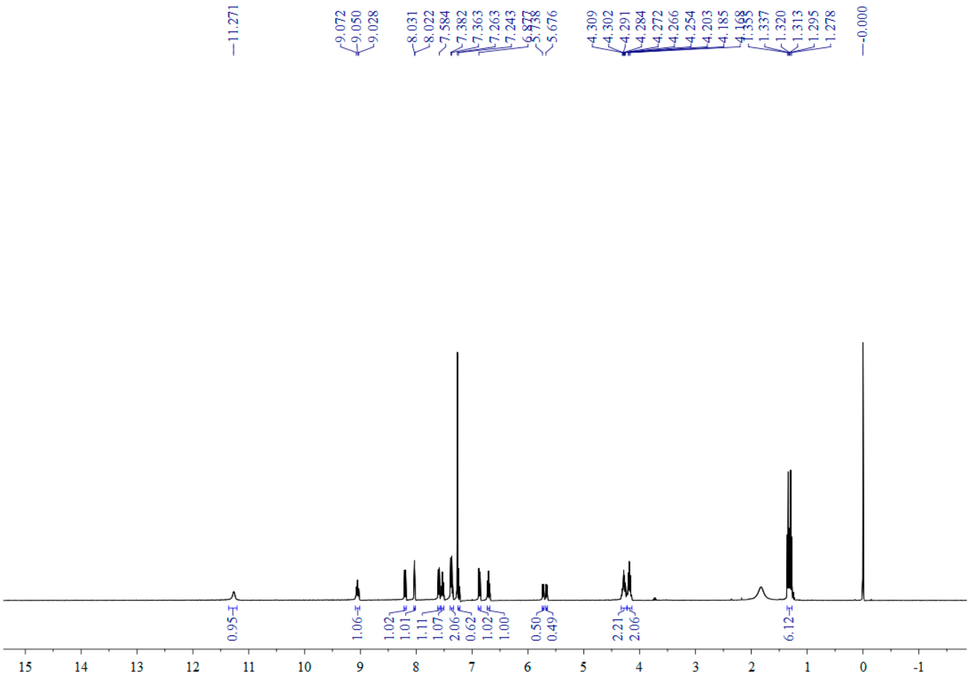

Figure S20. ^{1}H-NMR of compound **4g**.

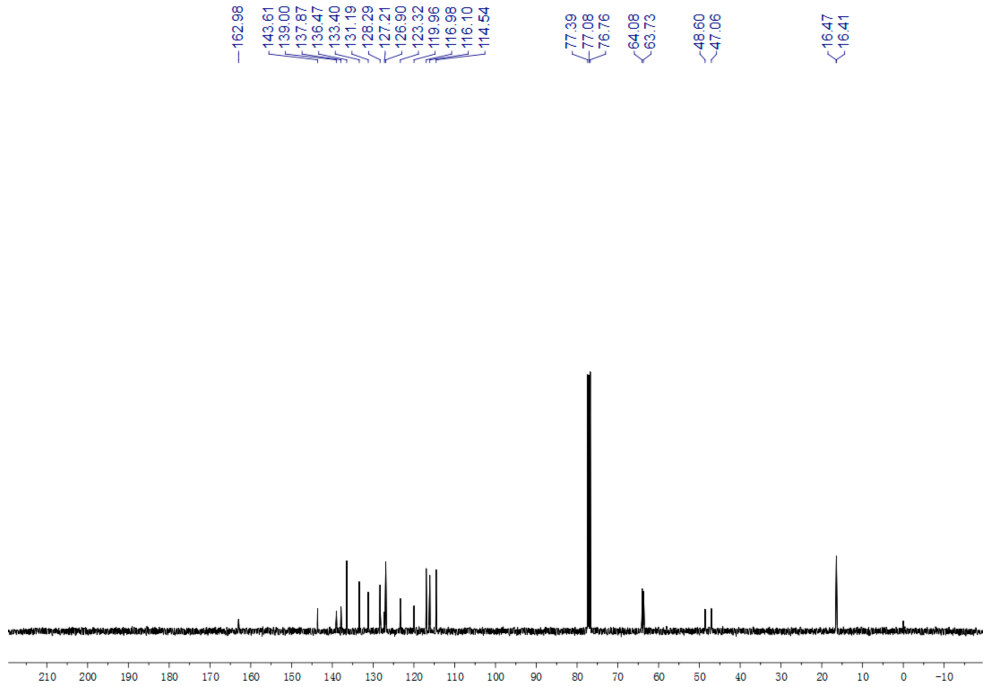

Figure S21. ^{13}C-NMR of compound **4g**.

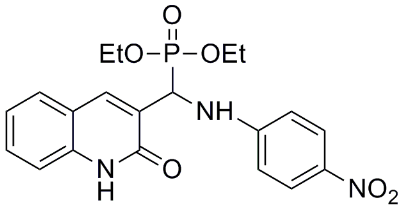

Figure S22. Chemical structure of compound **4h**.

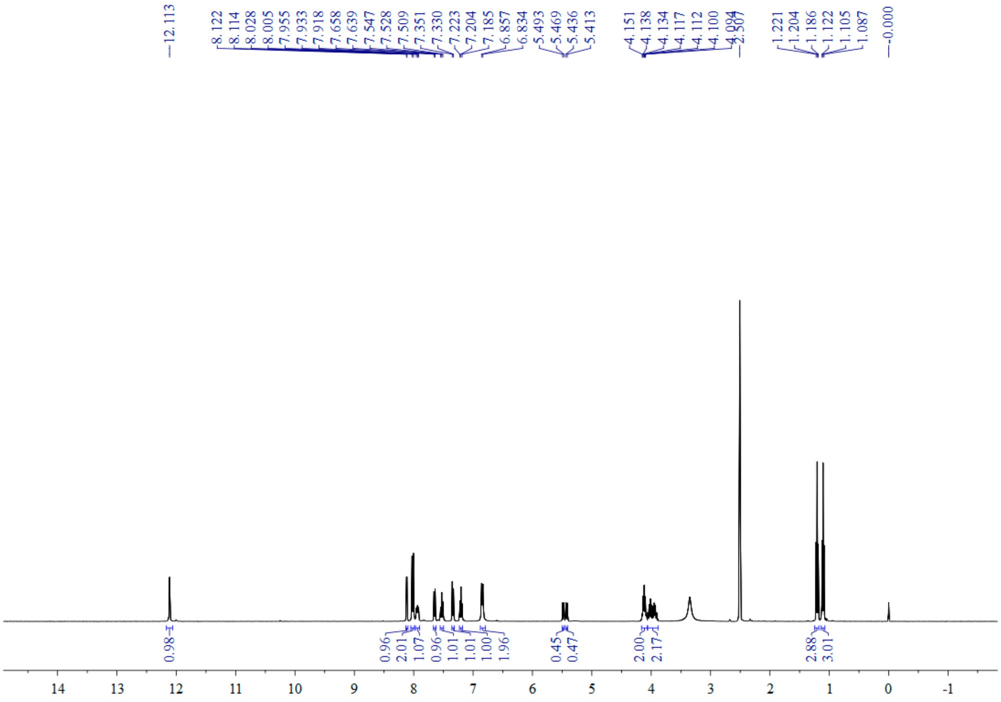

Figure S23. ^{1}H-NMR of compound **4h**.

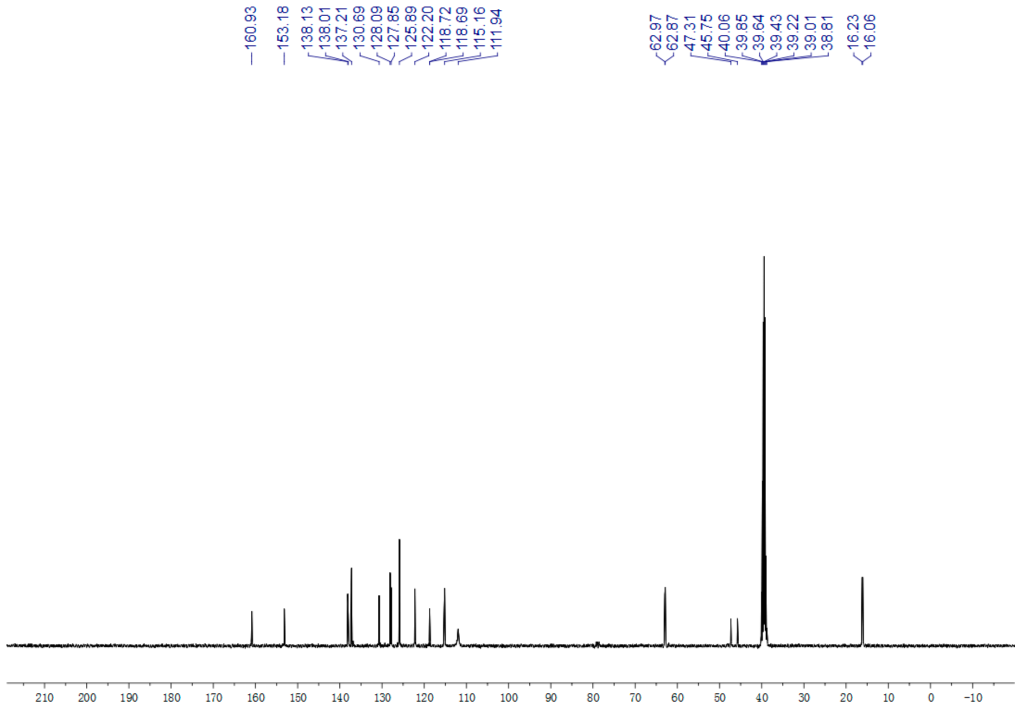

Figure S24. ^{13}C-NMR of compound **4h**.

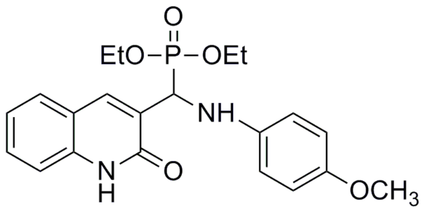

Figure S25. Chemical structure of compound **4i**.

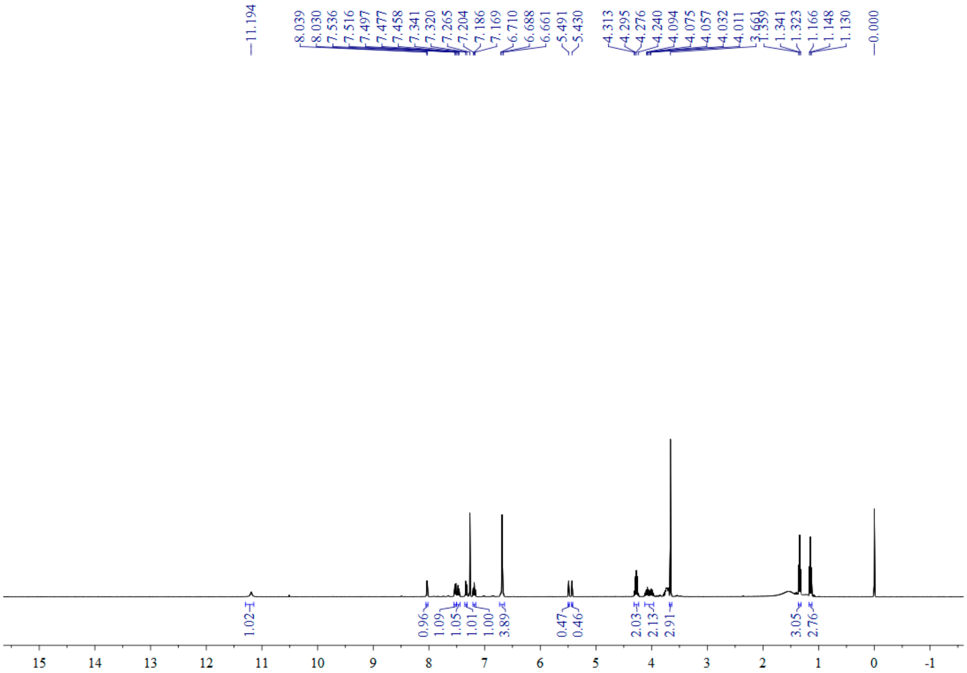

Figure S26. ^{1}H-NMR of compound **4i**.

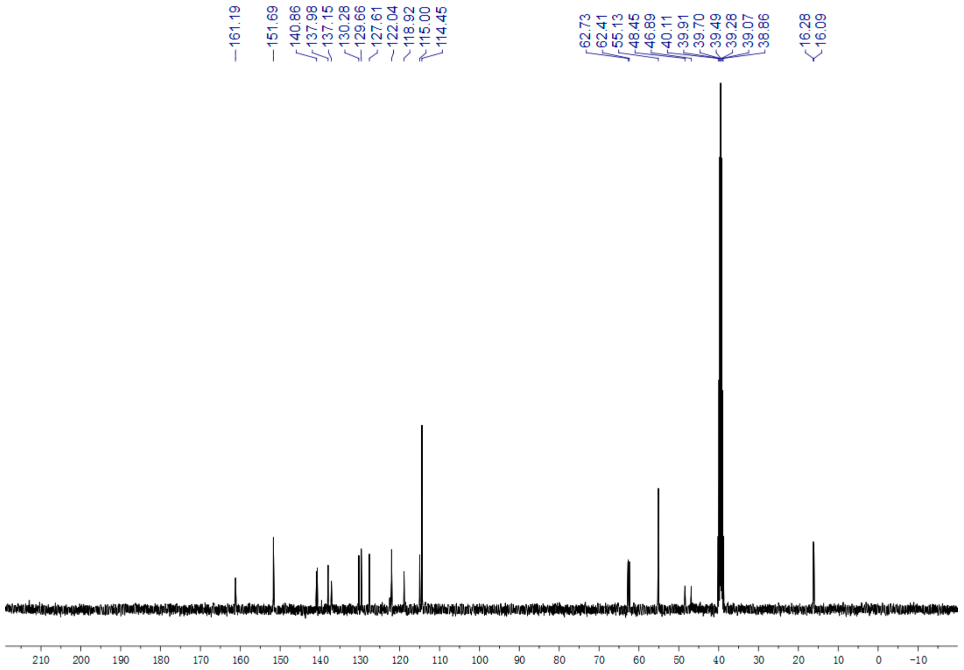

Figure S27. ^{13}C-NMR of compound **4i**.

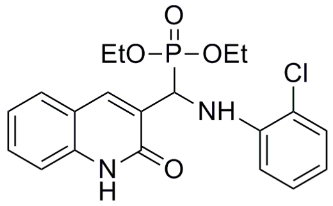

Figure S28. Chemical structure of compound **4j**.

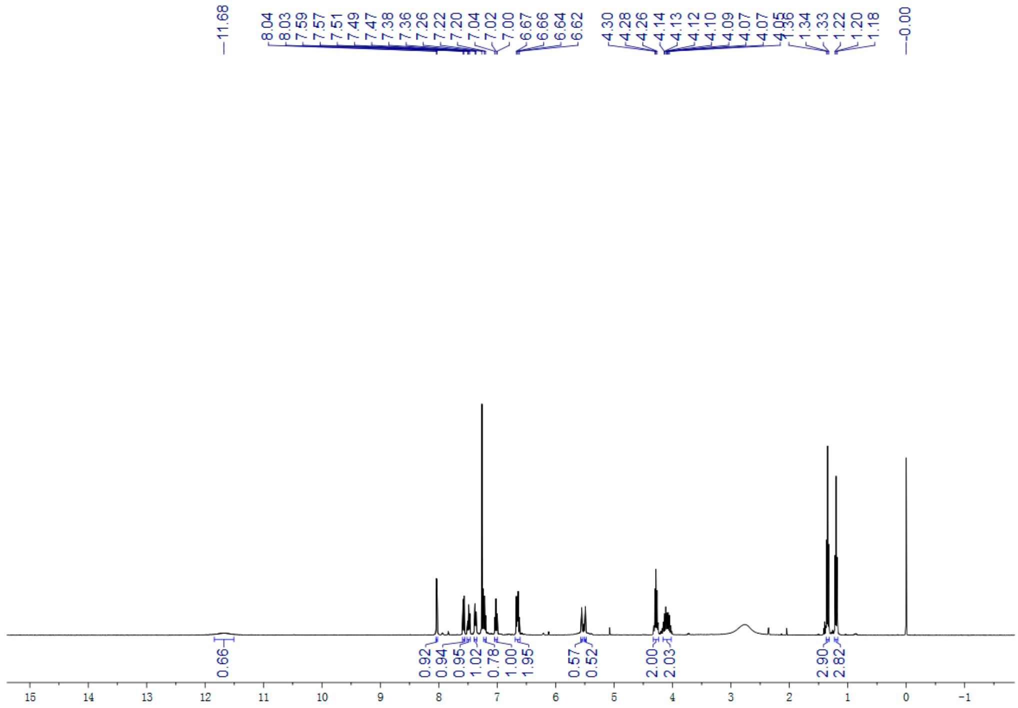

Figure S29. ^{1}H-NMR of compound **4j**.

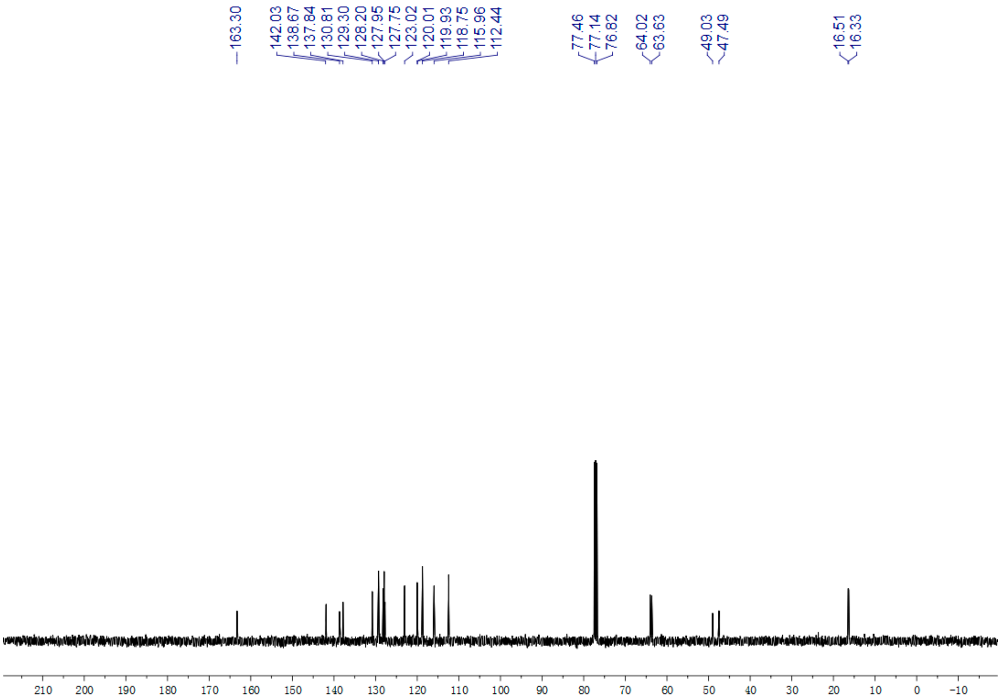

Figure S30. ^{13}C-NMR of compound **4j**.

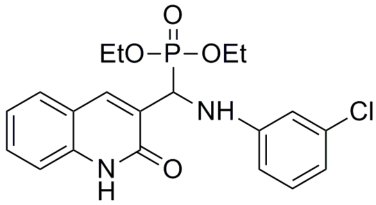

Figure S31. Chemical structure of compound **4k**.

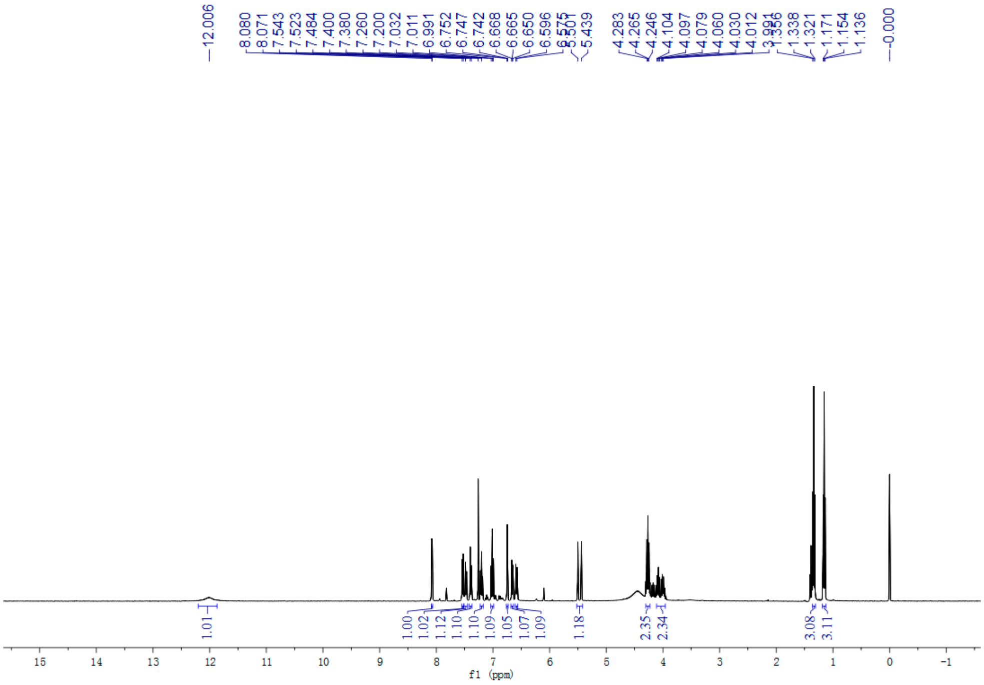

Figure S32. ^{1}H-NMR of compound **4k**.

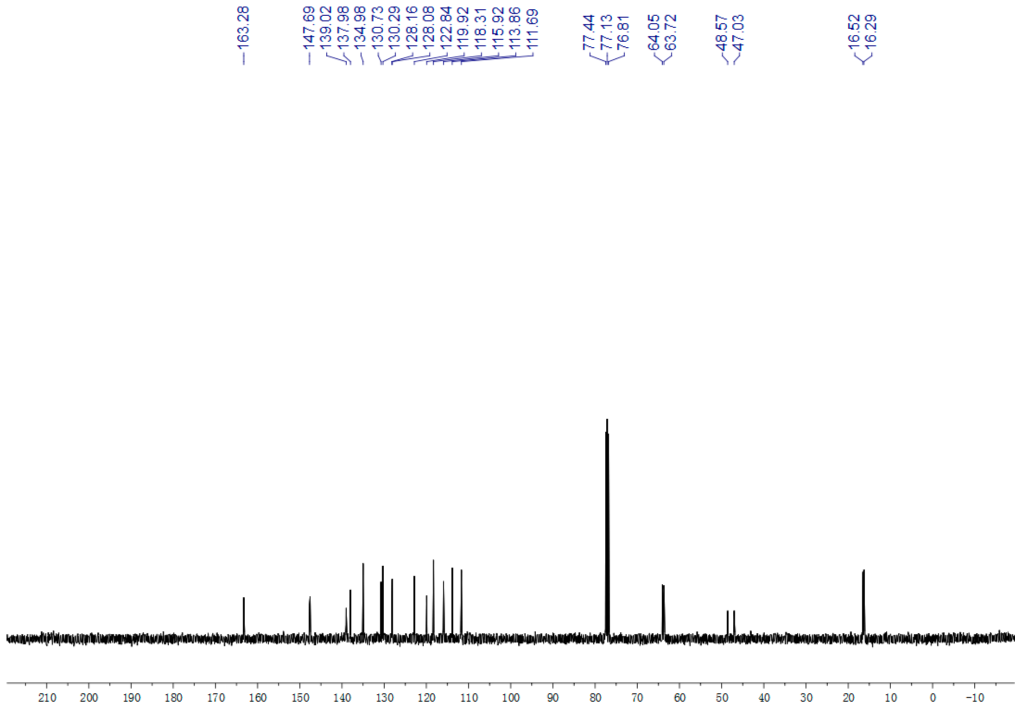

Figure S33. ^{13}C-NMR of compound **4k**.

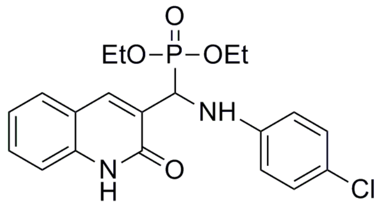

Figure S34. Chemical structure of compound **4l**.

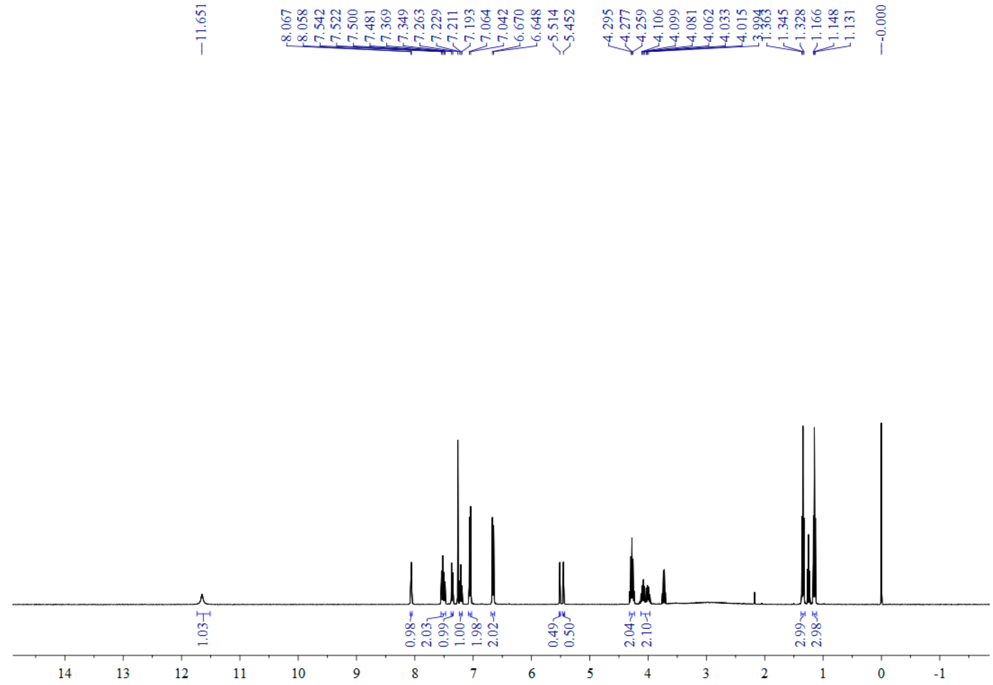

Figure S35. ^{1}H-NMR of compound **4l**.

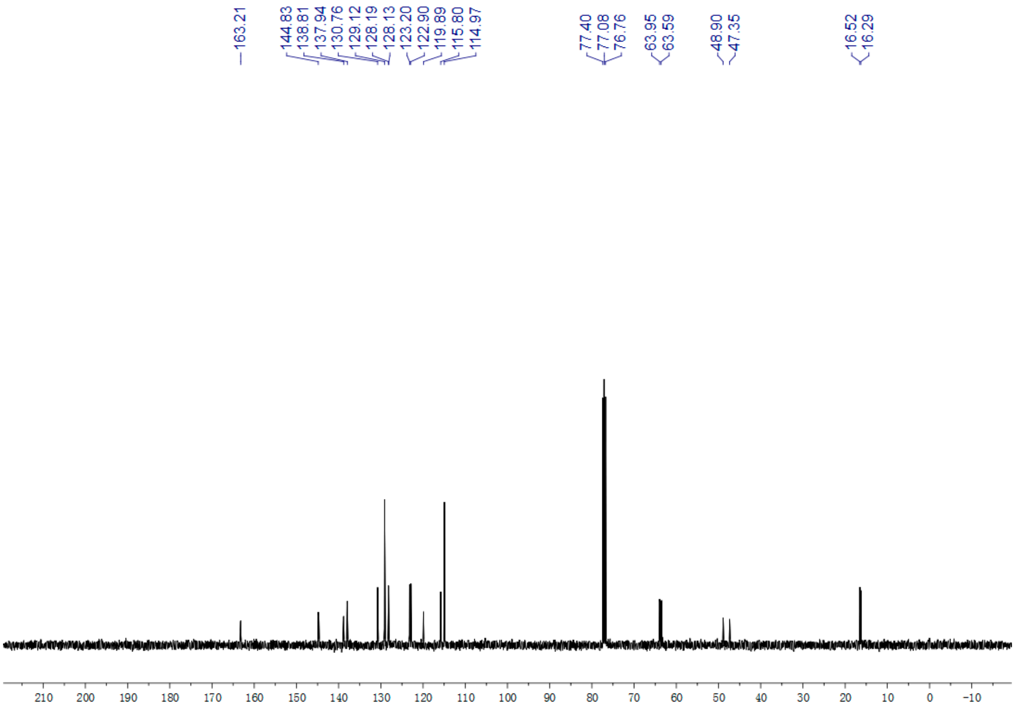

Figure S36. ^{13}C-NMR of compound **4l**.

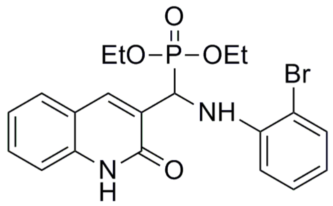

Figure S37. Chemical structure of compound **4m**.

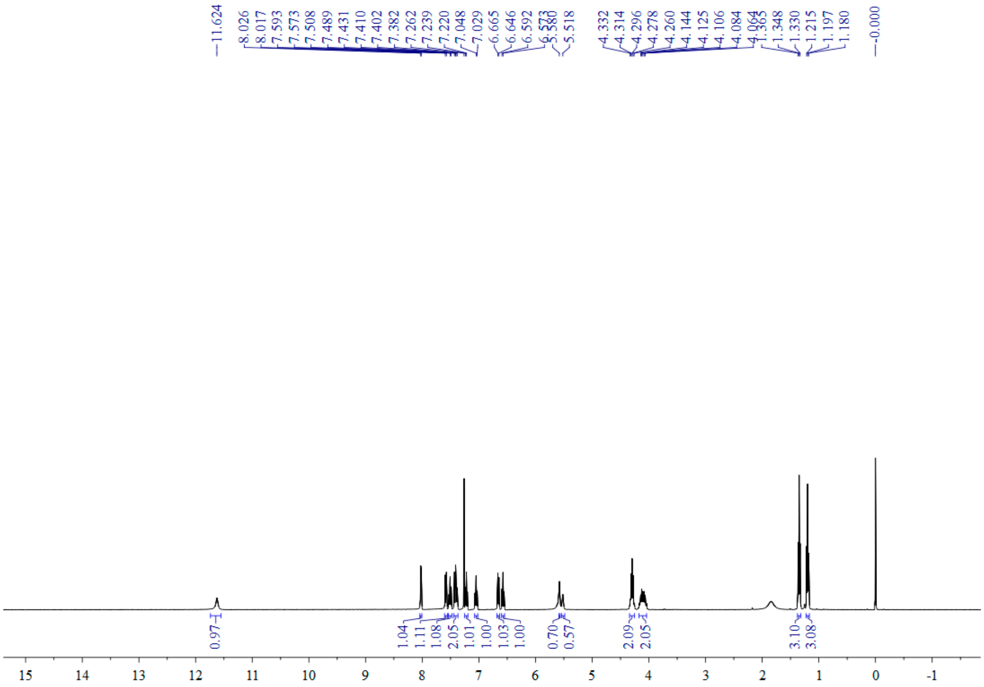

Figure S38. ^{1}H-NMR of compound **4m**.

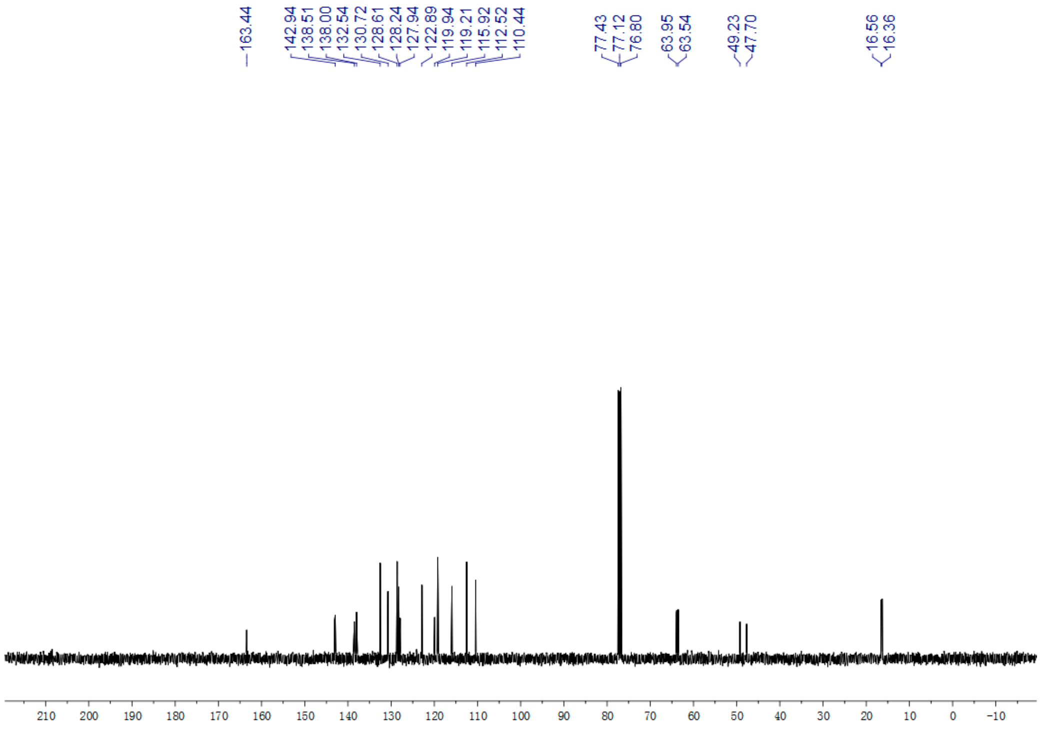

Figure S39. ^{13}C-NMR of compound **4m**.

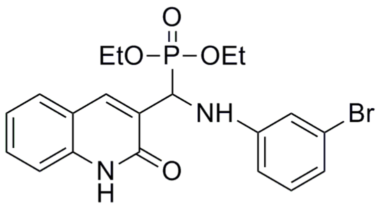

Figure S40. Chemical structure of compound **4n**.

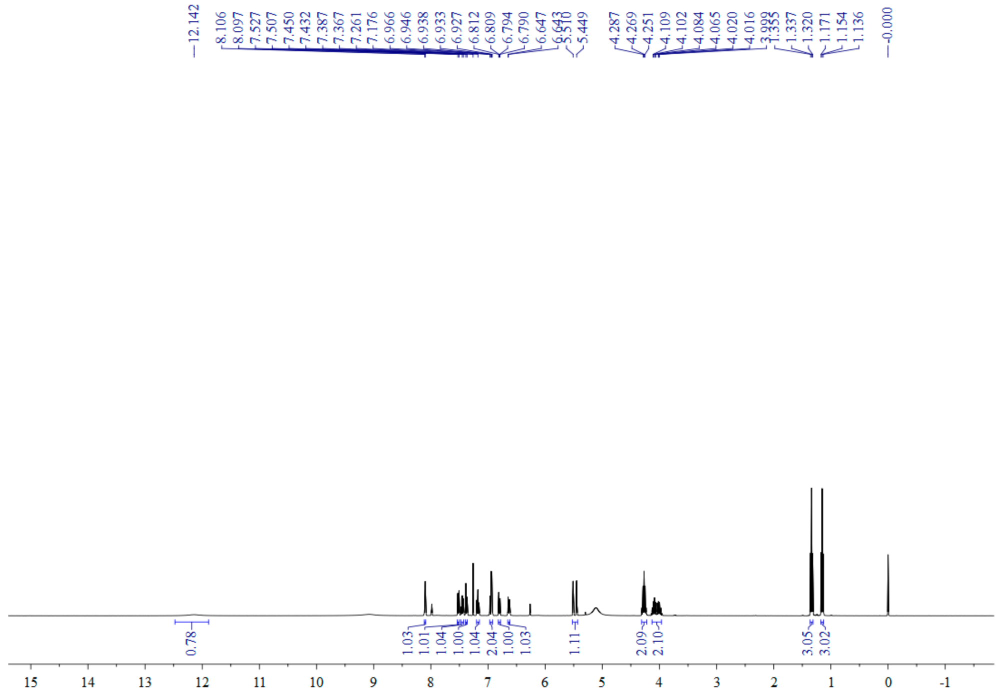

Figure S41. ^{1}H-NMR of compound **4n**.

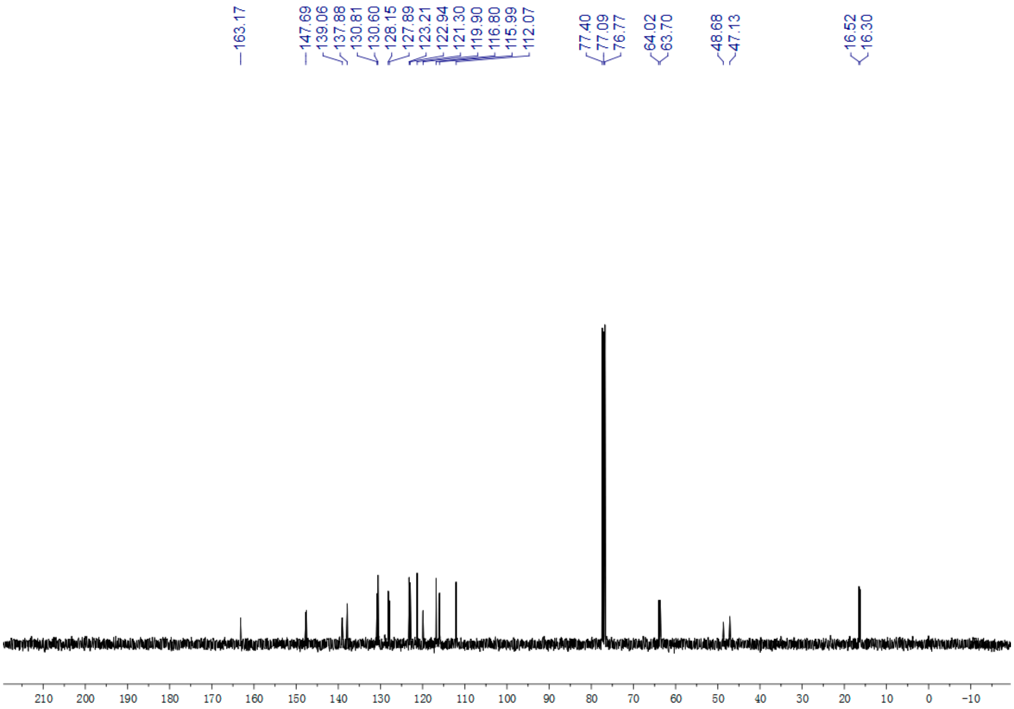

Figure S42. ^{13}C-NMR of compound **4n**.

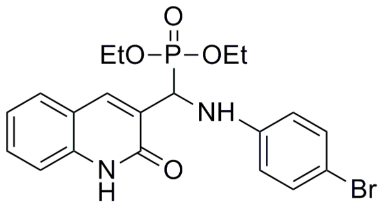

Figure S43. Chemical structure of compound **4o**.

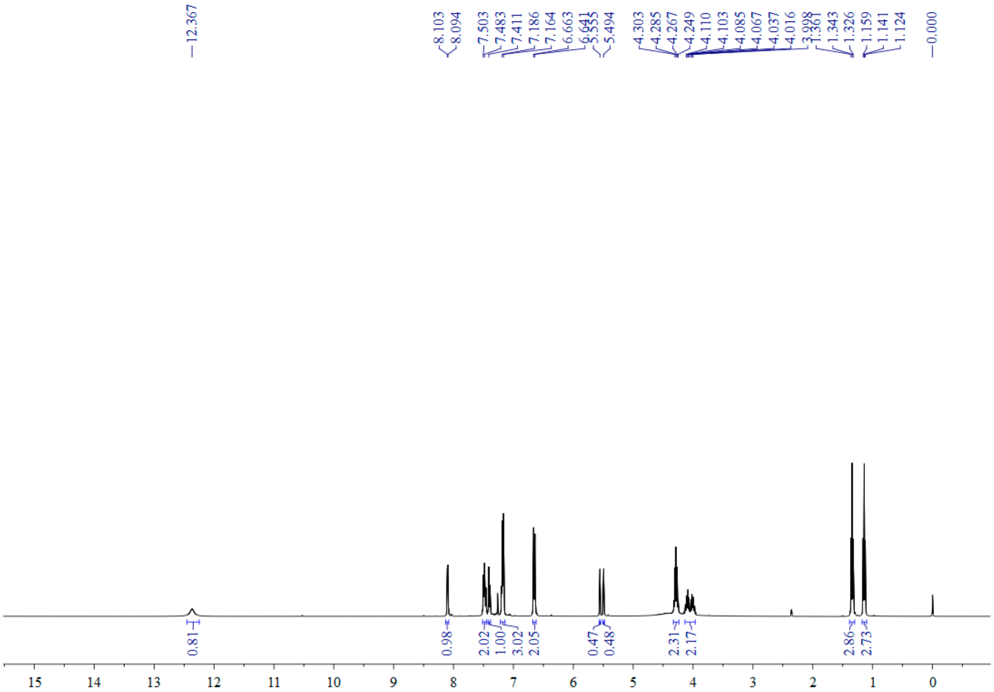

Figure S44. ^{1}H-NMR of compound **4o**.

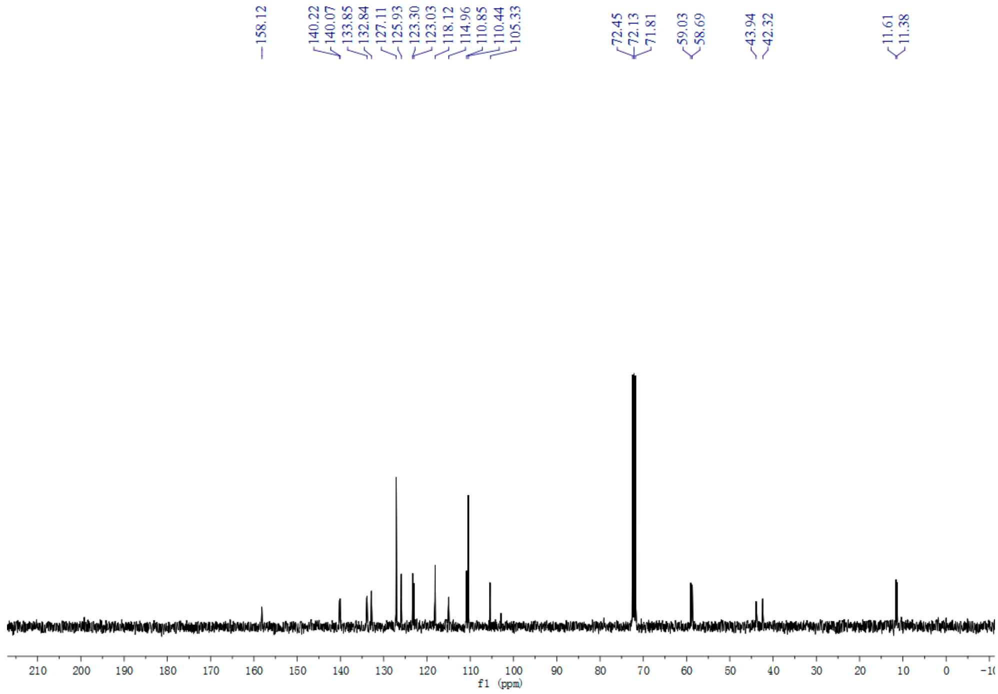

Figure S45. ^{13}C-NMR of compound **4o**.

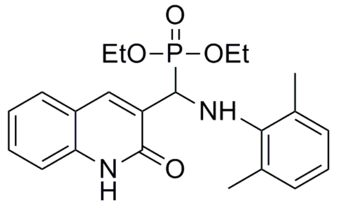

Figure S46. Chemical structure of compound **4p**.

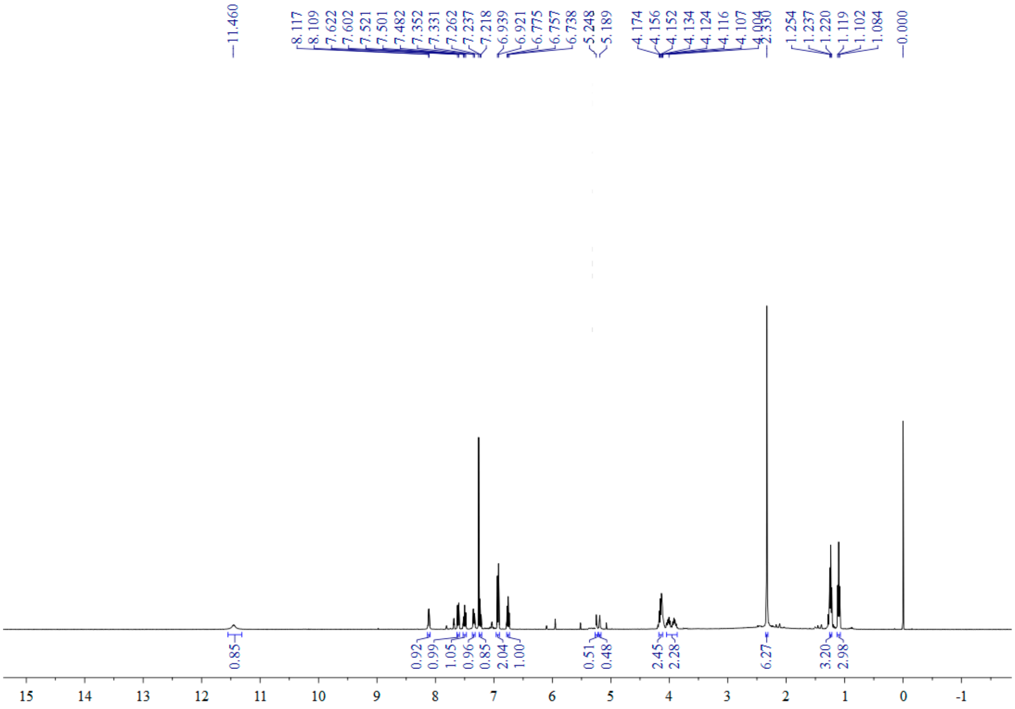

Figure S47. ^{1}H-NMR of compound **4p**.

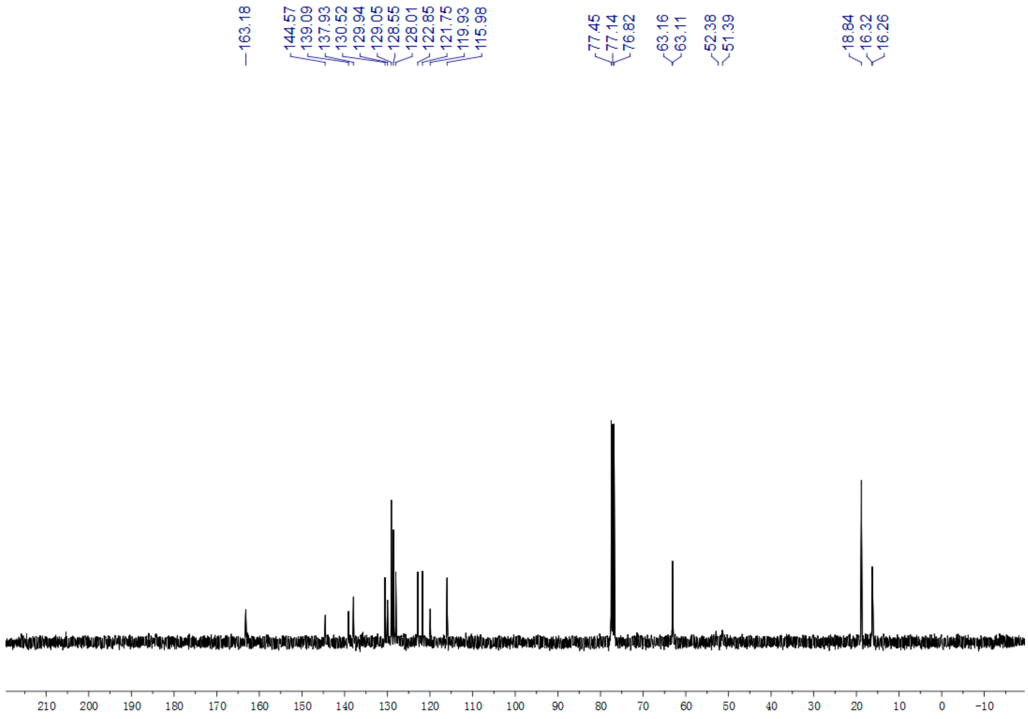

Figure S48. ^{13}C-NMR of compound **4p**.

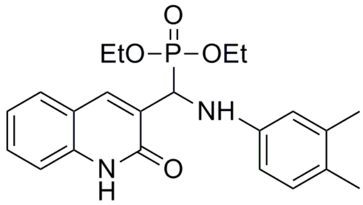

Figure S49. Chemical structure of compound **4q**.

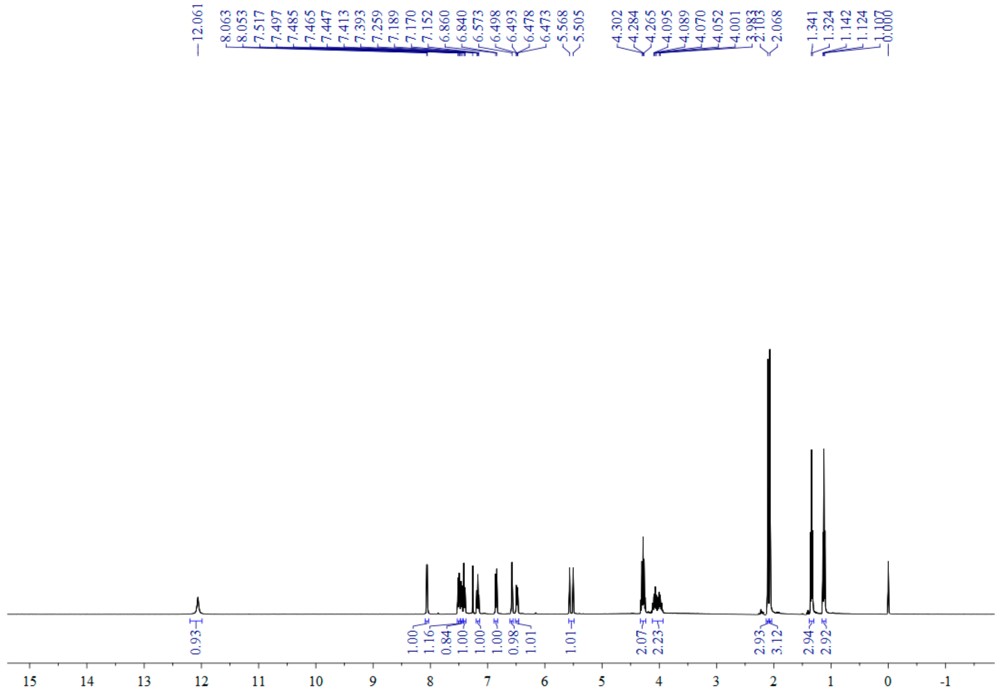

Figure S50. ^{1}H-NMR of compound **4q**.

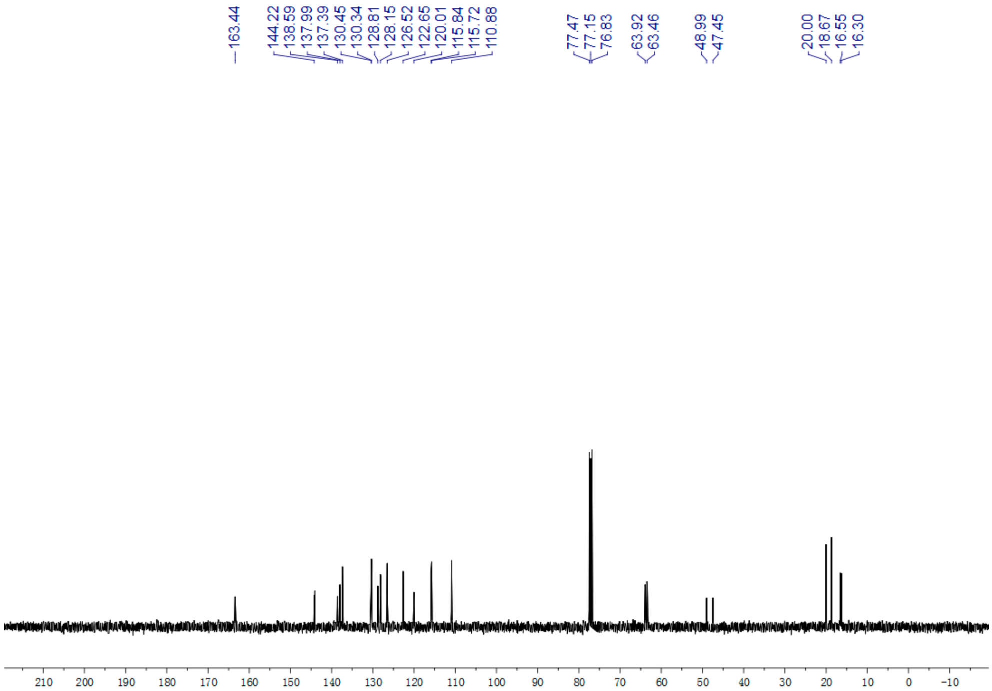

Figure S51. ^{13}C-NMR of compound **4q**.

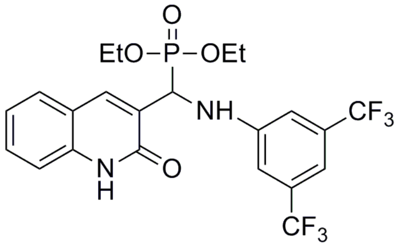

Figure S52. Chemical structure of compound **4r**.

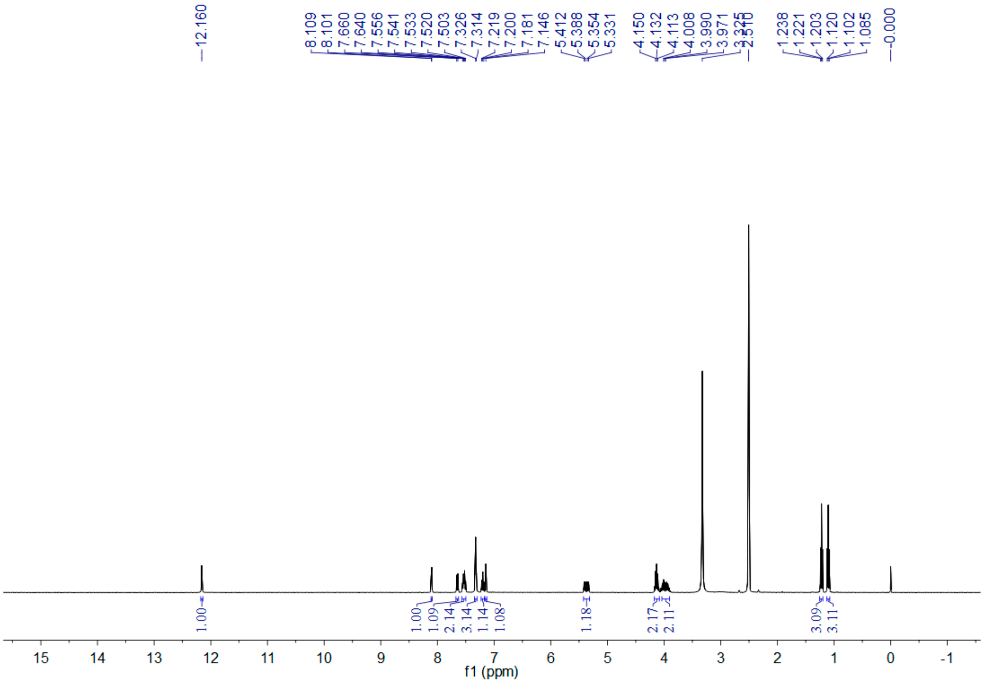

Figure S53. ^{1}H-NMR of compound **4r**.

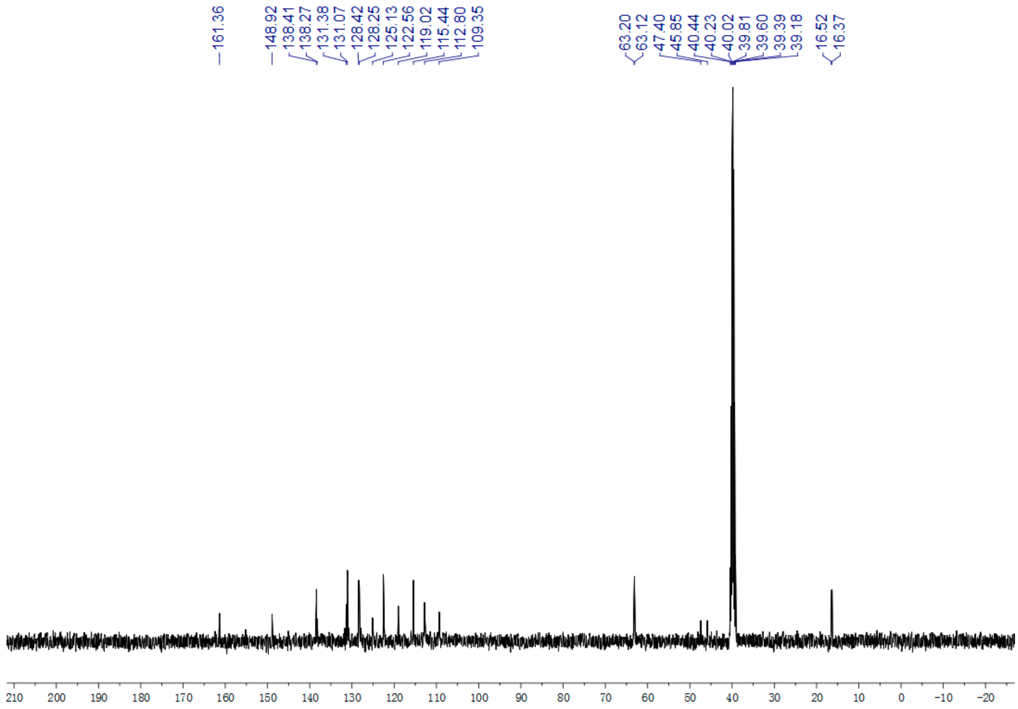

Figure S54. ^{13}C-NMR of compound **4r**.

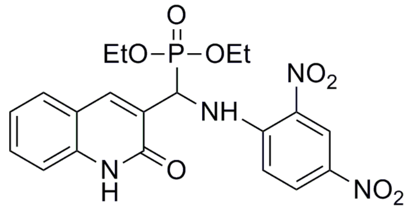

Figure S55. Chemical structure of compound **4s**.

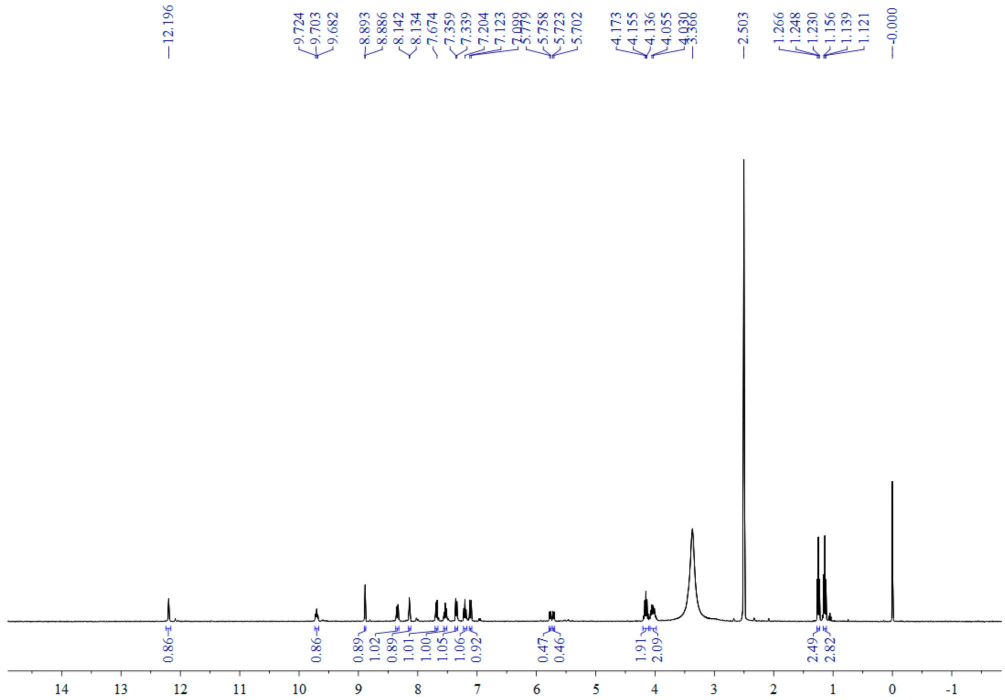

Figure S56. ^{1}H-NMR of compound **4s**.

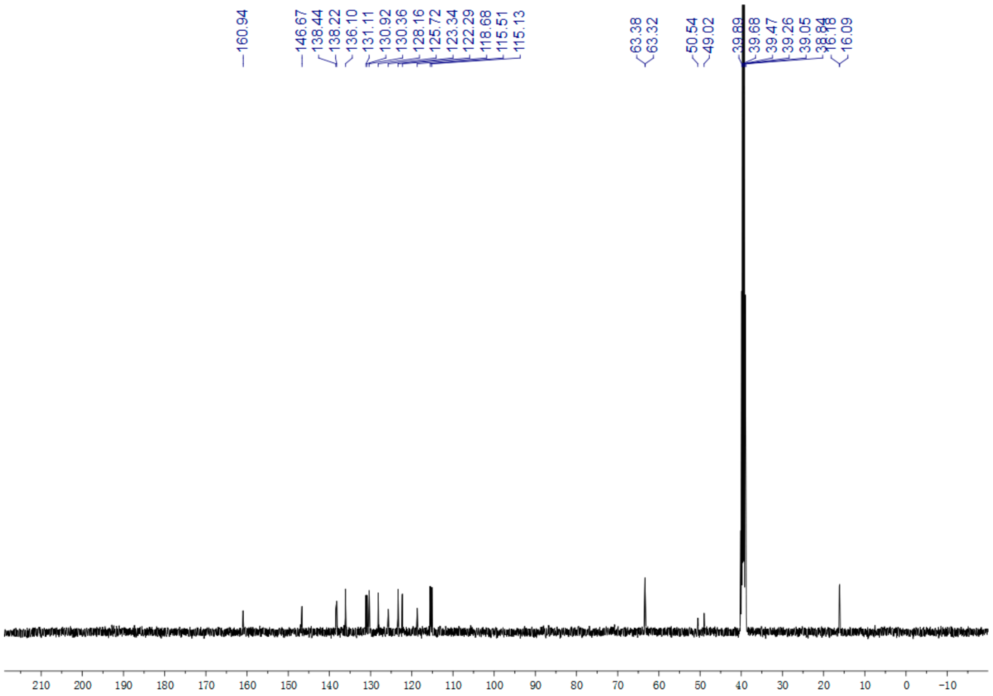

Figure S57. ^{13}C-NMR of compound **4s**.

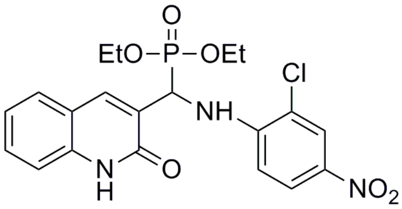

Figure S58. Chemical structure of compound **4t**.

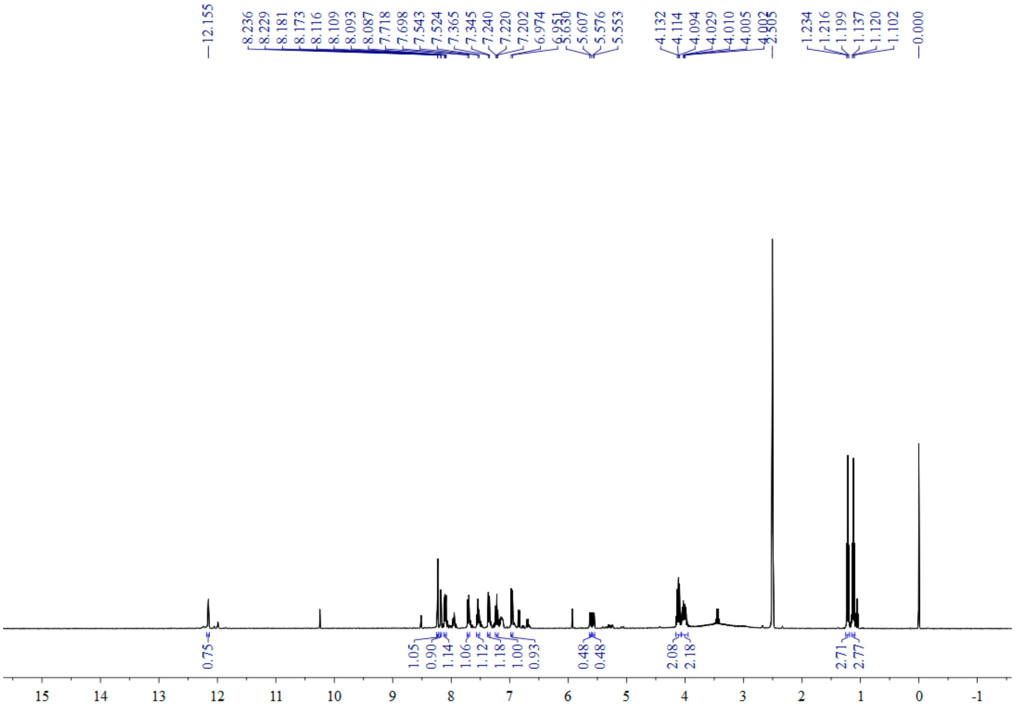

Figure S59. ^{1}H-NMR of compound **4t**.

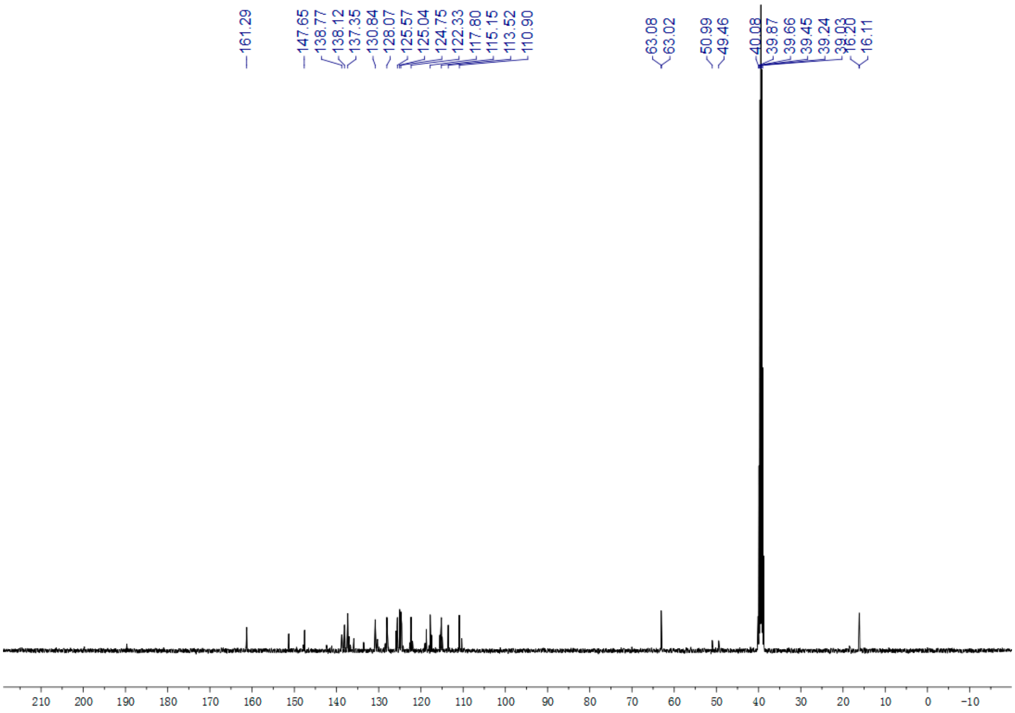

Figure S60. ^{13}C-NMR of compound **4t**.

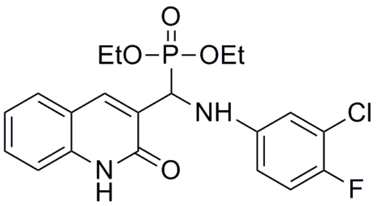

Figure S61. Chemical structure of compound **4u**.

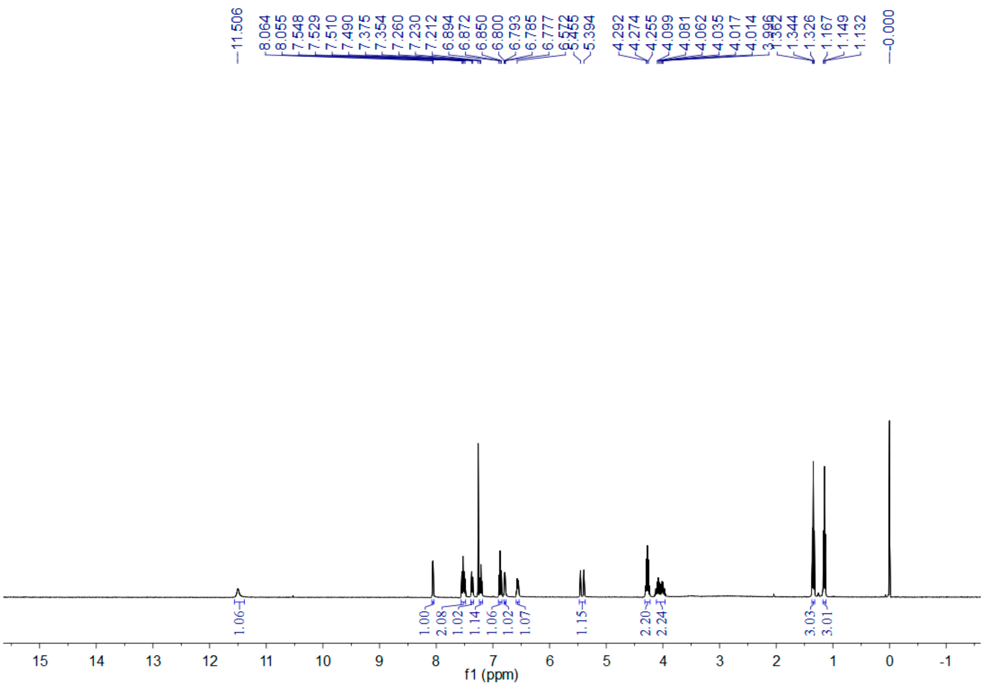

Figure S62. ^{1}H-NMR of compound **4u**.

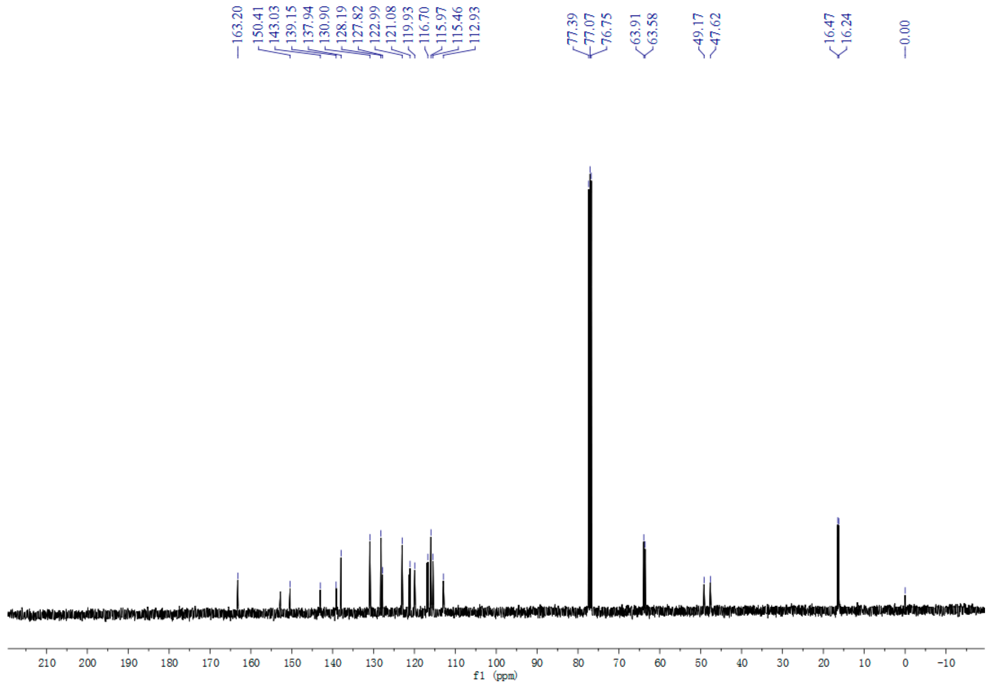

Figure S63. ^{13}C-NMR of compound **4u**.

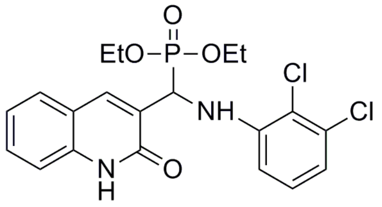

Figure S64. Chemical structure of compound **4v**.

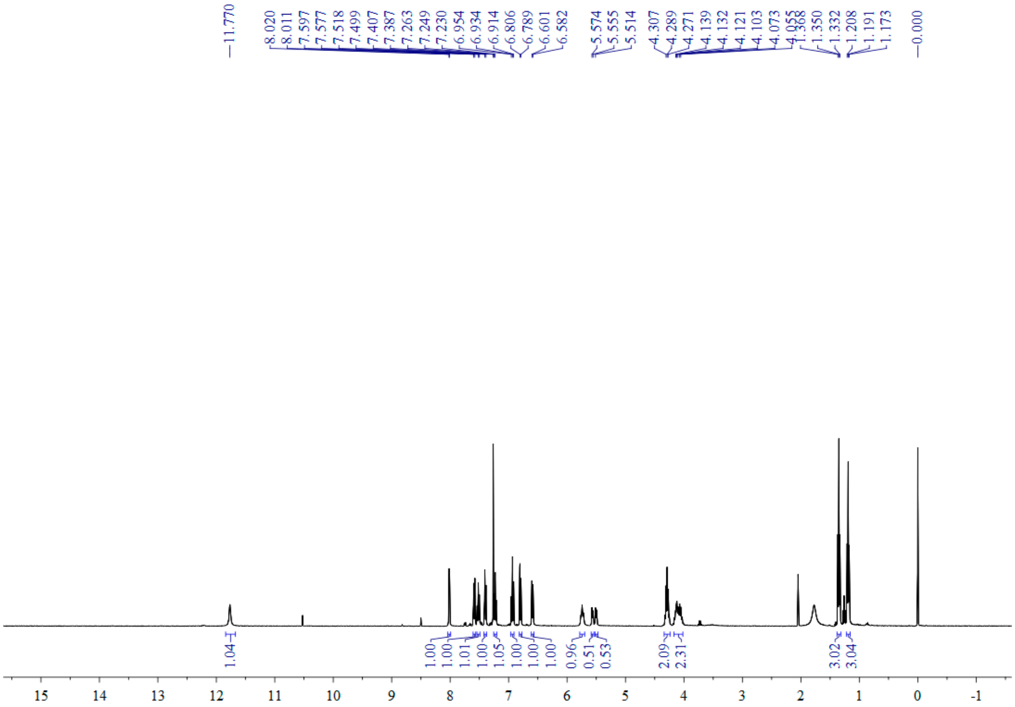

Figure S65. ^{1}H-NMR of compound **4v**.

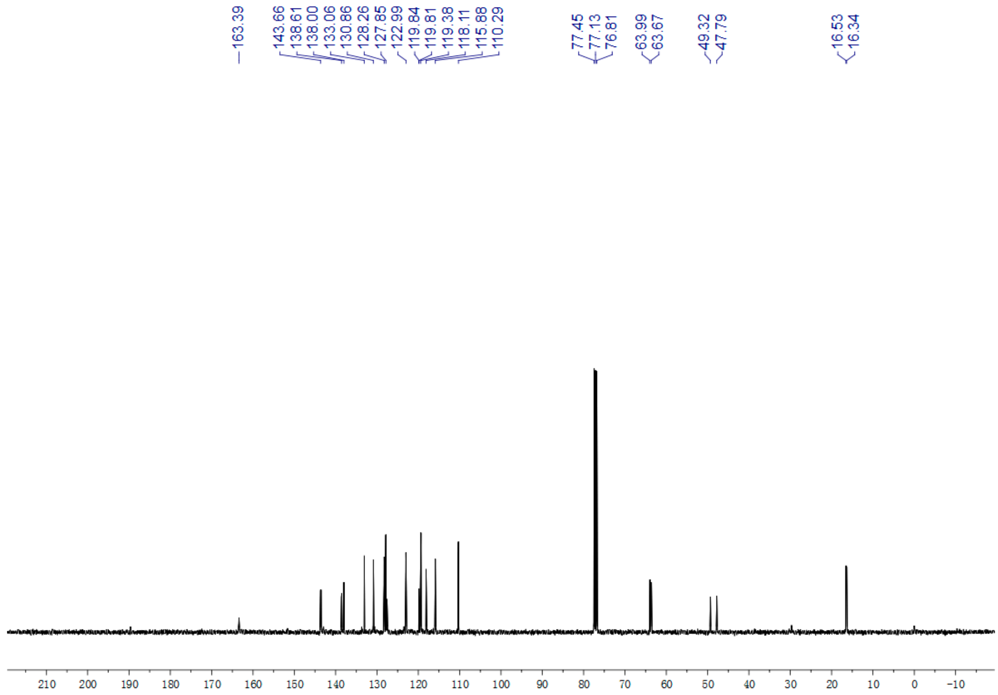

Figure S66. ^{13}C-NMR of compound **4v**.

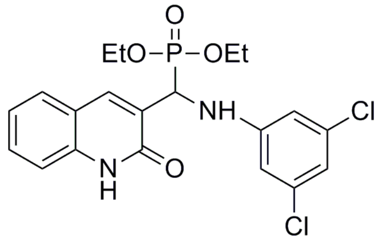

Figure S67. Chemical structure of compound **4w**.

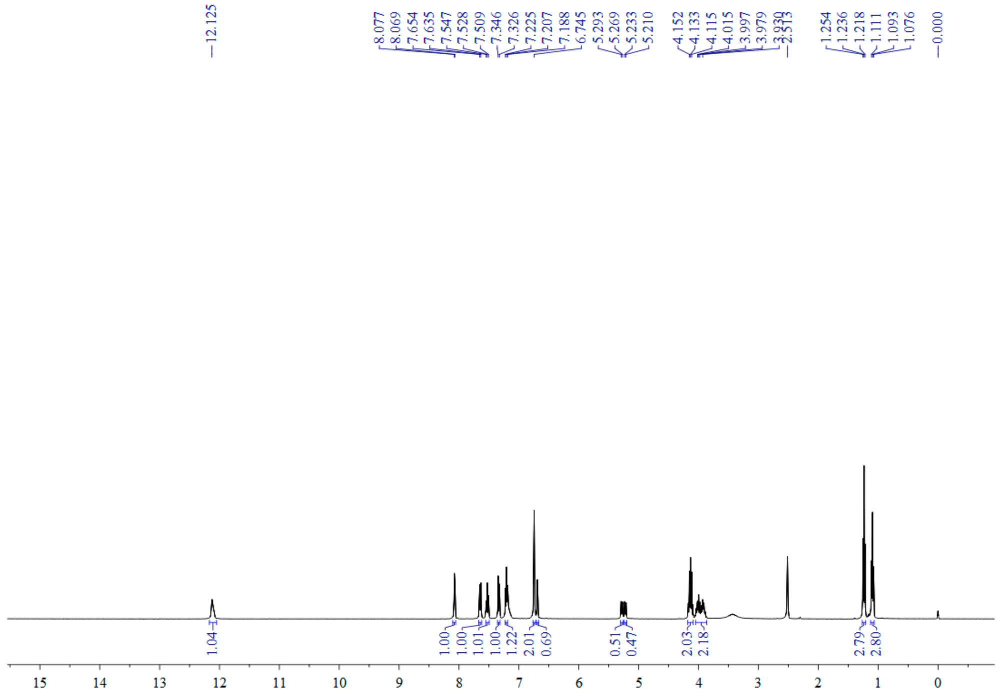

Figure S68. ^{1}H-NMR of compound **4w**.

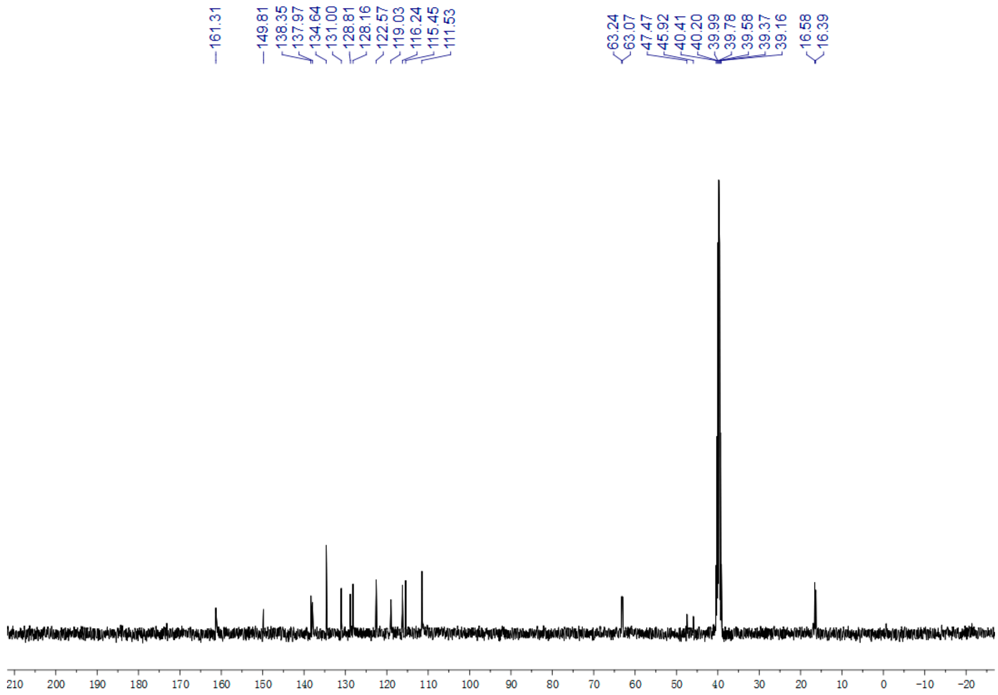

Figure S69. ^{13}C-NMR of compound **4w**.

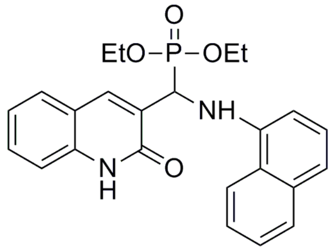

Figure S70. Chemical structure of compound **4x**.

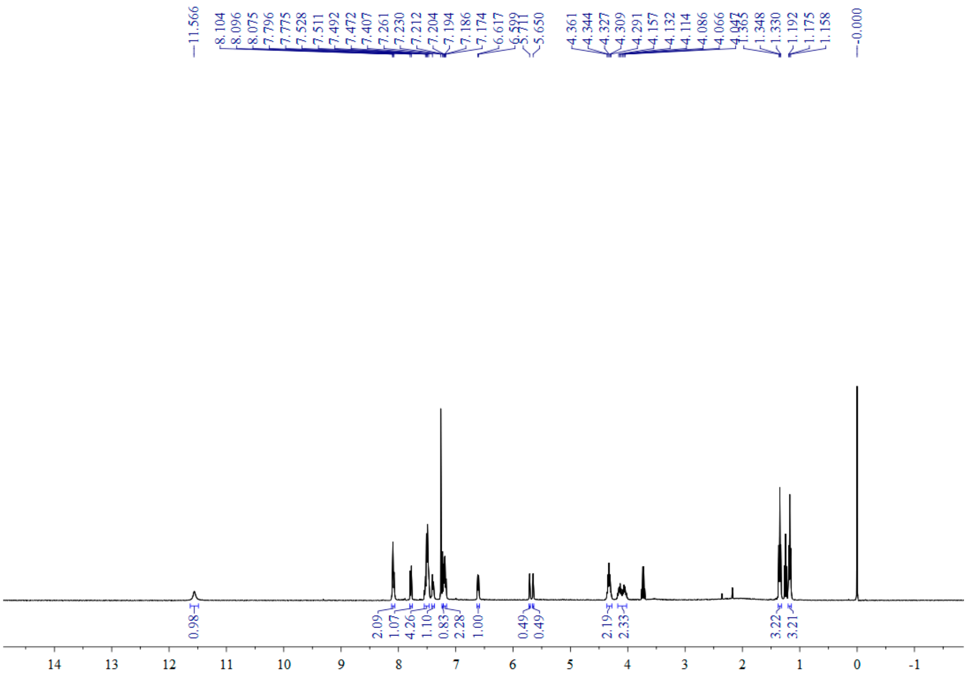

Figure S71. ^{1}H-NMR of compound **4x**.

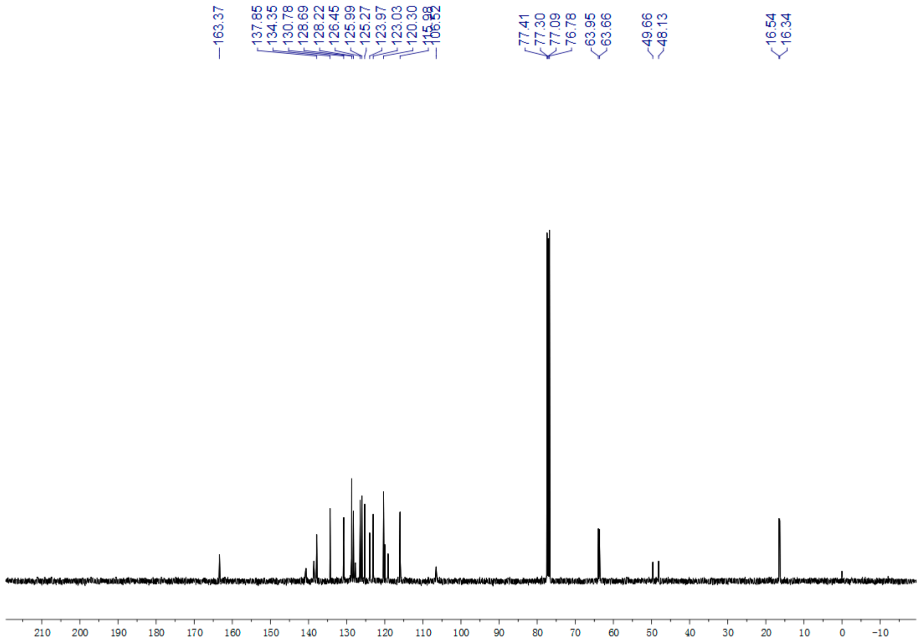

Figure S72. ^{13}C-NMR of compound **4x**.

© 2016 by the authors; licensee MDPI, Basel, Switzerland. This article is an open access article distributed under the terms and conditions of the Creative Commons by Attribution (CC-BY) license (http://creativecommons.org/licenses/by/4.0/).
